# Automated FAZ segmentation and diabetic retinopathy classification using OCTA images

**DOI:** 10.1186/s12886-025-04473-2

**Published:** 2025-10-28

**Authors:** Jamshid Saeidian, Hamid Riazi-Esfahani, Hossein Azimi, Hossein Farrokhpour, Ali Momeni, Mahdi Jamalitootakani, Ahmad Mirshahi, Hooshang Faghihi, Reza Sadeghi, Elias Khalili pour

**Affiliations:** 1 Faculty of Mathematical Sciences and Computer, Kharazmi University, No. 50, Taleghani Avenue, Tehran, Iran; 2 Retina Service, Farabi Eye Hospital, Tehran University of Medical Sciences, Box: 14176-13151, South Kargar Street, Qazvin Square, Qazvin Street, Tehran, Iran

**Keywords:** Diabetic retinopathy, Foveal avascular zone, Image segmentation, Deep learning, Classification, OCTA

## Abstract

**Background:**

Accurate segmentation of the foveal avascular zone (FAZ) is valuable for retinal imaging, as FAZ alterations are key biomarkers for diabetic retinopathy (DR). This study presents an automated framework exploring the feasibility of FAZ segmentation and DR classification using optical coherence tomography angiography (OCTA) images.

**Methods:**

In this cross-sectional study conducted at Farabi Eye Hospital, Tehran, Iran, a two-step deep learning pipeline was developed. First, a neural network combining DeepLabv3+, EfficientNetB0, Squeeze-and-Excitation (SE) blocks, and Atrous Spatial Pyramid Pooling (ASPP) was trained to segment the FAZ from superficial capillary plexus (SCP) and deep capillary plexus (DCP) OCTA slabs. Second, a GoogLeNet-based convolutional neural network (CNN) classified segmented FAZ images into binary (normal vs. DR) and three-class (normal, non-proliferative DR [NPDR], proliferative DR [PDR]) categories to differentiate DR stages based on FAZ shape characteristics. For the classification task using the deep learning-generated segmented FAZ images as input, the data was split into 70% training, 10% validation, and 20% testing, with 5-fold cross-validation to mitigate overfitting. Data augmentation and Synthetic Minority Oversampling Technique (SMOTE) were applied to improve classification performance.

**Results:**

The final dataset comprised 253 OCTA scans (126 SCP, 127 DCP) from 161 eyes of 161 participants (one eye per participant), with 39 normal participants (24.2%), 78 patients with NPDR (48.4%), and 44 with PDR (27.3%). The mean age was 49.7 ± 11.8 years, and 54% were male. The FAZ segmentation network achieved a Dice similarity coefficient (DSC) of 97.5% across the dataset, achieving high precision even in challenging images. The classification model, using the deep learning generated segmented FAZ images as input, reached an area under the curve (AUC) of 100% for binary classification (normal vs. DR) and 87% for three-class classification (normal, NPDR, PDR) with oversampling.

**Conclusion:**

This system, with its potential for integrating into clinical workflows, offers a promising assistive tool for clinicians, which could enable earlier and more accurate diagnosis of diabetic retinopathy from OCTA images.

**Clinical trial number:**

Not applicable.

**Supplementary Information:**

The online version contains supplementary material available at 10.1186/s12886-025-04473-2.

## Introduction

Diabetic Retinopathy (DR) is a progressive retinal disorder and a leading cause of vision loss in the working-age population, with an expected increase in burden over the next years, leading to an estimated 160 million individuals with DR by 2045 [1]. 

Retinal neovascularization, diabetic macular edema (DME), and diabetic macular ischemia (DMI) are the primary causes of vision loss during DR progression [2, 3]. Non-Proliferative Diabetic Retinopathy (NPDR) and Proliferative Diabetic Retinopathy (PDR) are two main classifications generally used for DR staging [4]. Differentiating PDR from NPDR can offer complementary information for guiding treatment and predicting disease progression. PDR, with its risk of severe complications like vitreous hemorrhage and retinal detachment, requires timely intervention such as laser therapy or anti-VEGF treatment [5]. In contrast, NPDR generally carries a lower risk of vision loss and is managed with regular monitoring and systemic control [6]. Early differentiation ensures appropriate management to preserve vision [4]. 

Optical coherence tomography angiography (OCTA) has emerged as an effective retinal imaging device, enabling non-invasive retinal microvasculature visualization at various depths, two features not available in fluorescein angiography (FA) [7]. Considering the importance of the macular region on vision, various imaging biomarkers in identifying macular alterations in DR and their use for diagnostic and prognostic implications have been characterized [8–11]. 

The foveal avascular zone (FAZ), a small capillary-free elliptical or circular area with well-defined borders in the central part of the retina, is crucial for high-acuity vision. It has been established as a key biomarker for assessing macular ischemia in various diseases, including DR [12]. Given the FAZ enlargement and irregularity development in its boundaries before and during DR progression, various OCTA-derived metrics have been generated to quantify FAZ morphology features, including FAZ area, perimeter, axis ratio, acircularity index, and roundness [13–15]. Several studies have demonstrated that FAZ alterations are not only correlated with DR severity and visual function [16–19] but also enhance the predictive accuracy of models for determining progression in longitudinal studies [18–21]. 

Manual FAZ segmentation, the first step in FAZ analysis, is time-consuming, resource-intensive, subjective, and prone to human error. This is particularly true when delineating FAZs with irregular, faded, or ‘moth-eaten’ borders, which are common in DR [3, 10]. This has led to high inter-observer variability, particularly in the deep capillary plexus [22–26]. 

To overcome these challenges, various studies have presented automated techniques for precise FAZ segmentation [27–38]. (Online Resource 1- Table A) In recent years, deep learning-based approaches, particularly Convolutional Neural Networks (CNNs), have shown great promise in FAZ segmentation in DR [39–44]. Automatic learning of hierarchical features from raw image data has allowed CNNs to detect complex patterns in FAZ morphology.

Despite these advantages, some aspects remain elusive. While overall segmentation accuracies have been reported, the efficacy of deep learning-based FAZ segmentation across each category, particularly in more challenging groups like PDR, has not been explored. Using FA, which offers complementary information for DR staging in particular stages, could improve classification robustness. Additionally, few studies have evaluated the potential of directly using segmented FAZ images in classifying DR stages as input for another deep learning model, instead of relying on quantified FAZ metrics.

To address the aforementioned fields, in this feasibility study, we propose two neural network-based approaches to first automate the segmentation of the FAZ from OCTA images across three classifications of normal, NPDR, and PDR, using advanced techniques, including the Squeeze and Excitation Network (SE-Block), EfficientNetB0, DeepLabv3+, and Atrous Spatial Pyramid Pooling (ASPP). Then, another deep learning model was developed to evaluate the performance of the deep learning segmented FAZ images for predicting whether eyes had DR and their staging, utilizing transfer learning with GoogLeNet. Finally, the performance of these models was assessed using appropriate metrics.

## Methods

### Study population and dataset

This retrospective study was conducted at Farabi Eye Hospital, Tehran University of Medical Sciences, Iran, from 2022 to 2023, adhering to the Declaration of Helsinki and approved by the institutional review board (IR.TUMS.FARABIH.REC.1401.034). Written informed consent was obtained from all participants.

Patients with DR and healthy controls were included, with DR diagnosed via retinal fundus examinations and FA (Heidelberg Engineering, Heidelberg, Germany) by two retina experts (H.R.E. and E.K.P.) with consensus. A senior physician resolved disagreements. Patients with neovascularization elsewhere (NVE) or at the disc (NVD) were labeled PDR; others were NPDR. Patients with NPDR were further classified as mild, moderate, and severe. Exclusion criteria included uncontrolled glaucoma, uveitis, severe media opacity, high refractive error (<-3 or > + 3), macular diseases, visual acuity < 20/200, history of intravitreal anti-VEGF, macular or panretinal photocoagulation, or intraocular surgery (except cataract surgery). Images with a signal strength index (SSI) < 40 were excluded.

OCTA imaging used the RTVue XR 100 Avanti device (Version 2017.1.0.151, Optovue, Inc., Fremont, CA, USA) with 3 × 3 mm scans centered on the macula, acquired between 8:00 a.m. and 12:00 a.m. Superficial capillary plexus (SCP; from internal limiting membrane [ILM] to 9 μm above the inner plexiform layer [IPL]) and deep capillary plexus (DCP; 9 μm above IPL to 9 μm below outer plexiform layer [OPL]) slabs were generated. Manual FAZ annotations, including irregular margins and areas, were performed by two experts (H.R.E. and E.K.P.) using ImageJ software. The inter-expert agreement, examined by Dice score, was 98.5%. The agreement was assessed on 120 stratified-random OCTA scans with 60 SCP, 60 DCP; one image per patient, and class proportions matched to the cohort. The ground truth manual segmentation was defined as the consensus between the two retinal specialists.

Although in the healthy control group, the OCTA images retained the Early Treatment Diabetic Retinopathy Study (ETDRS) grid overlay from the device’s default output, the FAZ region was entirely enclosed within the central 1 mm circle, and the grid lines did not interfere with the FAZ boundary and segmentation. This overlay was not present in the DR cohort.

**Fig. 1:**
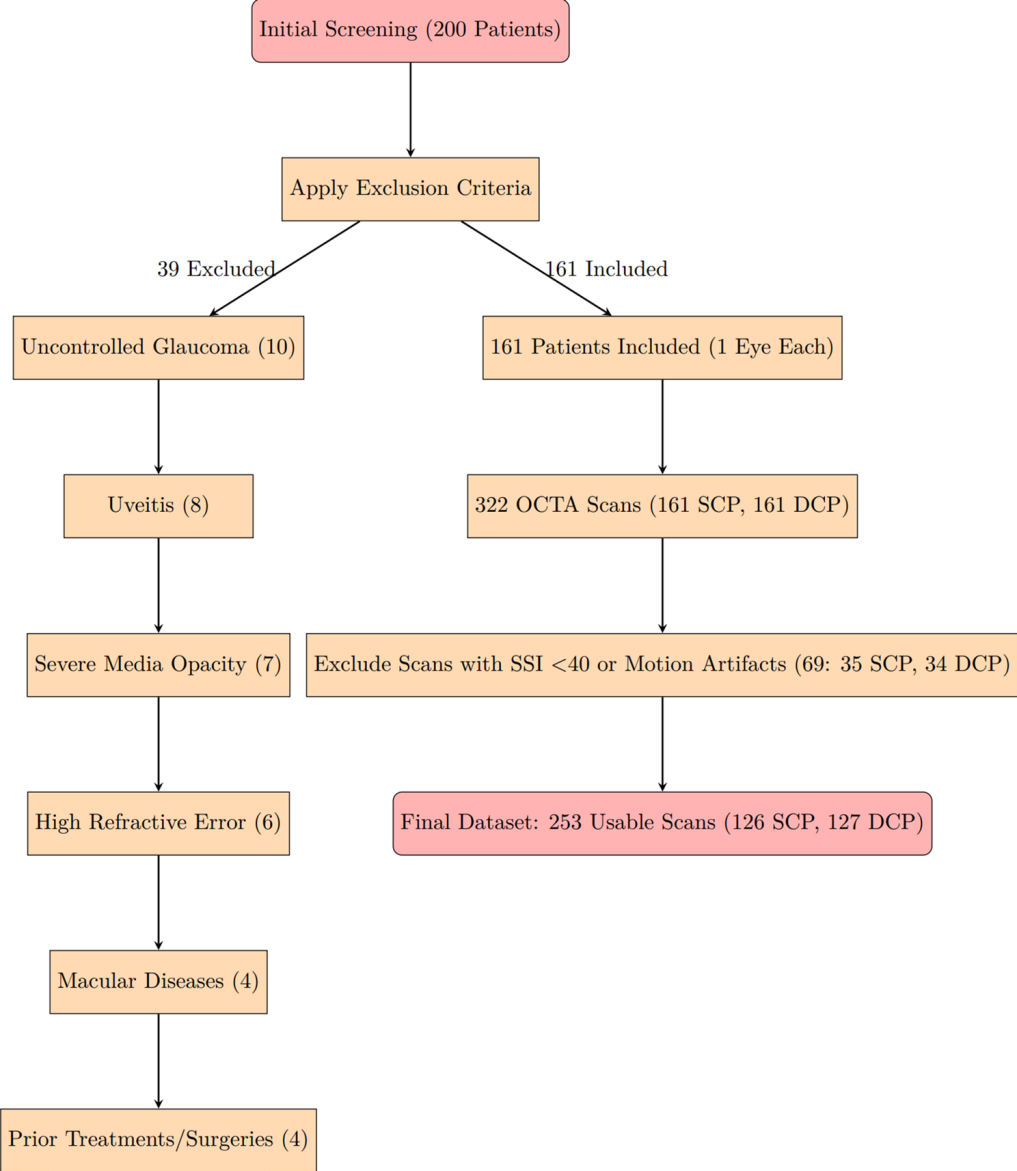
Flowchart detailing patient inclusion and exclusion. Initially, 200 patients were screened; 39 were excluded due to uncontrolled glaucoma (10), uveitis (8), severe media opacity (7), high refractive error (6), macular diseases (4), or prior treatments/surgeries (4). Of 161 included participants, 253 scans (126 superficial capillary plexus (SCP), 127 deep capillary plexus (DCP)) were usable after excluding 69 scans due to signal strength index (SSI) < 40 or motion artifacts

The dataset comprised 253 OCTA scans from 161 participants (one eye per participant): 39 normal participants (24.2%), 78 participants with NPDR (48.4%; 23 with mild NPDR, 27 with moderate, and 28 with severe), and 44 individuals with PDR (27.3%). After excluding images with SSI < 40 or motion artifacts, 126 SCP and 127 DCP scans remained (Fig. 1). Demographic and clinical details are in Table 1.

### Image preprocessing

The preprocessing steps included denoising, normalization, and resizing. To address noise artifacts commonly present in OCTA images, we applied the Non-Local Means (NLM) denoising method. By leveraging the redundancy of similar pixels within the image, the NLM algorithm computes a weighted average of neighboring pixels, leading to smoother images without significant loss of information. Following the denoising process, we performed image normalization to ensure that the pixel intensity values across all images were standardized. This step involved scaling the pixel values to a range of [0, 1] to ensure consistency across the dataset. Finally, to maintain uniformity across the dataset, we resized all images to dimensions of 352 × 352 pixels. Images of varying sizes were adjusted using appropriate interpolation techniques, known as spline interpolation. This approach was chosen because conventional resizing methods often result in the loss of fine details, particularly around edges and corners. By using spline interpolation, these details can be preserved to a much greater extent. This ensured that the aspect ratio was preserved as much as possible and that the critical features within the images remained recognizable.

### Image segmentation network development

The segmentation phase of our study focused on the automated extraction of the FAZ from OCTA images using a deep-learning approach. Our architecture combines previously published advanced techniques, including the adoption of the DeepLabv3 + segmentation framework with its built-in Atrous Spatial Pyramid Pooling (ASPP) feature [45], combined with EfficientNetB0 as the backbone [46] and Squeeze and Excitation Network (SE-Block) [47], as additional components, to achieve high accuracy and robustness in segmentation. Below, we elaborate on each component and justify its inclusion in the network.

Prior to finalizing the proposed architecture, we evaluated multiple segmentation networks to identify the most effective configuration for FAZ extraction. The Dice Similarity Coefficient (DSC) achieved by U-Net, LinkNet, pyramid scene parsing network (PSPNet), UNet++, and DeepLabv3 + was 84.0%, 81.2%, 83.6%, 87.6%, and 92.4%, respectively. Incorporating EfficientNetB0 and SE-Blocks into the DeepLabv3 + framework further improved the DSC to 97.5%.

#### Squeeze and excitation network

The SE-Block enhances the representational power of neural networks by emphasizing relevant features and suppressing irrelevant ones [47]. (Online Resource 1- Figure A) By learning channel-wise feature dependencies, SE-Blocks enable the network to focus on significant aspects of the input image, such as the intricate vascular structures surrounding the FAZ. Advantages of SE-Block included improving feature recalibration, leading to better focus on target regions like the FAZ, enhancing the network’s ability to generalize across different image conditions and complexities, and minimizing computational overhead while significantly boosting performance. In our network, SE-Blocks were integrated after convolutional layers to ensure that critical features related to FAZ delineation were prioritized, thereby improving segmentation outcomes.

#### EfficientNetB0

EfficientNetB0 was chosen as the backbone for our segmentation network due to its superior balance between performance and computational efficiency, its lightweight architecture, and its versatility for complex medical images [46]. (Online Resource 1- Figure B)

EfficientNetB0 provided high-level feature representations that were integrated into the subsequent segmentation layers of the network. We further fine-tuned it for our model. (Online Resource 1- Table B)

#### Atrous spatial pyramid pooling

The ASPP block is a core component of the DeepLabv3 + architecture [45]. It uses atrous convolutions with varying dilation rates to capture multi-scale features, enabling the network to focus on both local and global contexts simultaneously. In our implementation, the ASPP block utilized dilation rates of [1, 6, 12, 18] to extract features at various resolutions. (Online Resource 1- Figure C) This ensured comprehensive coverage of the FAZ region while maintaining computational efficiency.

The ASPP mechanism was specifically deployed on the high-level image features obtained from the sixth layer of the EfficientNetB0 architecture, which was selected as the pretrained model for this research. This strategic implementation aimed to effectively capture the multi-scale characteristics associated with varying sizes of the FAZ.

#### DeepLabv3 + network architecture

DeepLabv3 + is a state-of-the-art semantic segmentation architecture that extends the capabilities of DeepLabv3 by incorporating a decoder module [45]. This architecture is designed to refine segmentation outputs, particularly around object boundaries, which is critical for accurately capturing the FAZ region.

**Fig. 2:**
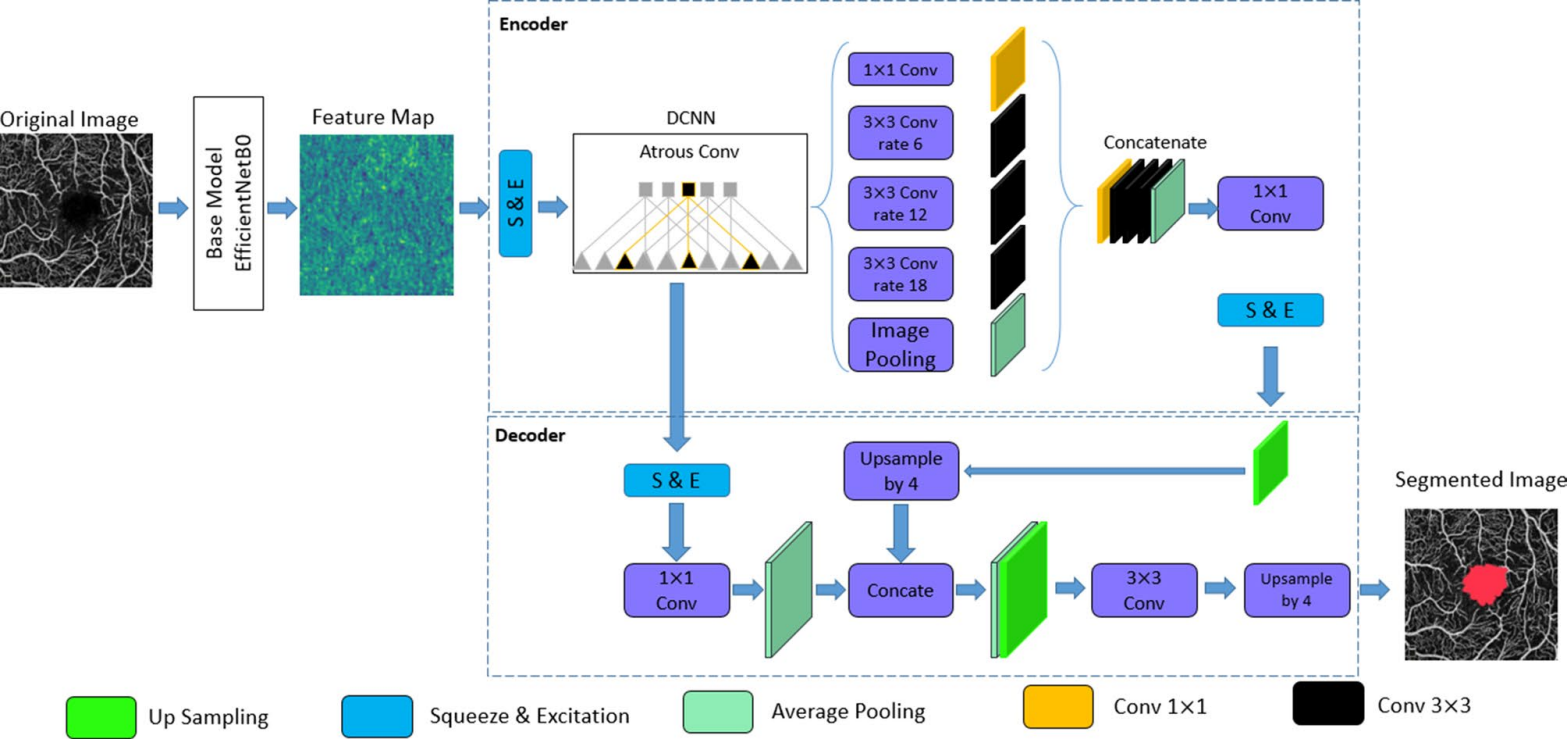
DeepLabv3 + Network Architecture. This schematic illustrates the DeepLabv3 + network architecture, a deep learning model for semantic segmentation tasks. DeepLabv3 + incorporates an encoder-decoder structure, where the encoder extracts features and reduces dimensionality, while the decoder refines segmentation results, particularly at object boundaries. To expand the receptive field while preserving feature map resolution, atrous convolutions are employed in the later stages of the encoder. The Atrous Spatial Pyramid Pooling (ASPP) module, also utilizing atrous convolutions with varying dilation rates, captures multiscale semantic context information

DeepLabv3 + was selected for its ability to handle the intricate boundaries of the FAZ region, ensuring detailed and accurate segmentation. These architectural improvements significantly enhance DeepLabv3 + SE’s capacity to deliver accurate semantic segmentation across a wide range of datasets (Fig. 2).

### Classification network development

The input to the classification model was segmentation-derived FAZ images generated by the DeepLabv3 + customized network. Classification used GoogLeNet with transfer learning, fine-tuned for binary (normal vs. DR) and three-class (normal, NPDR, PDR) tasks based on segmented FAZ images (Online Resource 1: Table C). We selected the model since its Inception branches (parallel 1 × 1, 3 × 3, 5 × 5 filters) provide multi-scale receptive fields well suited to FAZ shape/irregularity without unnecessary over-parameterization. Its parameter efficiency (~ 6.8 M weights) also mitigates overfitting in our single-center dataset (161 patients, 253 OCTA scans). By contrast, ResNet-50 (~ 25.6 M) and Vision Transformers (≥ 85 M) are substantially heavier and typically require larger-scale pretraining to perform reliably on smaller medical imaging tasks. Training was conducted on Kaggle using an NVIDIA RTX 3090 GPU with 24 GB memory. Patient data was processed locally, and only anonymized segmentation-derived FAZ images were used on Kaggle, with institutional approval for data handling.

### Model training, evaluation, and implementation

The dataset was split as follows: for segmentation, 80% (202 scans) for training, 20% (51 scans) for testing; for classification, 70% (177 scans) for training, 10% (25 scans) for validation, and 20% (51 scans) for testing, as illustrated in Table 2. The same 20% test set was used for both segmentation and classification tasks to ensure consistency. To address overfitting concerns, 5-fold cross-validation was implemented. The segmentation model used the ADAM optimizer (learning rate 0.001, reduced by 0.1 if validation loss plateaued for 10 epochs) with early stopping after 20 epochs. The classification model used the Synthetic Minority Oversampling Technique (SMOTE) to balance classes; [48] we used two techniques: oversampling, where minority classes were synthetically oversampled using SMOTE, until each class reached the target count. Arbitrary sampling, in which a balanced training set was created at the specified fixed size per class by combining random under-sampling of the majority classes and SMOTE oversampling of the minority classes as needed.

Segmentation was evaluated using intersection over union (IoU) and DSC. DCP vs. SCP segmentation metrics were compared within eyes using two-sided paired t-tests (normality checked by Shapiro–Wilk); Classification was assessed using AUC, sensitivity, specificity, and F1-score.

## Results

The dataset comprised 253 OCTA scans from 161 participants, consisting of 39 normal individuals (24.2%), 78 individuals with NPDR (48.4%), and 44 individuals with PDR (27.3%). Among the NPDR group, 23 cases had mild NPDR (29.5%), 27 cases had moderate NPDR (34.6%), and 28 cases had severe NPDR (35.9%), indicating that 70.5% of NPDR cases were moderate to severe. Demographic and clinical details are provided in Table 1. No notable differences in age or sex were detected (*P* > 0.05, ANOVA, chi-square).

**Table 1:** Participant demographics and clinical characteristics

Characteristic	Normal (*n* = 39)	NPDR (*n* = 78)	PDR (*n* = 44)	*p*-value *
Age (years, mean ± SD)	41.8 ± 7.2	52.3 ± 13.5	51.9 ± 8.6	0.87
Sex (% male)	53.8%	55.1%	52.3%	0.94
BCVA (logMAR)	0.05 ± 0.1	0.12 ± 0.15	0.18 ± 0.2	< 0.001
FAZ Area SCP (mm²)	0.25 ± 0.05	0.38 ± 0.08	0.52 ± 0.12	< 0.001
FAZ Area DCP (mm²)	0.28 ± 0.06	0.42 ± 0.09	0.58 ± 0.14	< 0.001

*: *P*-values calculated using ANOVA (continuous) or chi-square (categorical) tests. BCVA: best corrected visual acuity, DCP: deep capillary plexus, FAZ: foveal avascular zone, NPDR: non-proliferative diabetic retinopathy, PDR: proliferative diabetic retinopathy, SCP: superficial capillary plexus

**Table 2:** Dataset splits for Training, Validation, and testing

Class	Total eyes	Training (70%)	Validation (10%)	Testing (20%)
Normal	39	27	4	8
NPDR	78	55	8	15
PDR	44	31	4	9
Total	161	113	16	32

NPDR: non-proliferative diabetic retinopathy, PDR: proliferative diabetic retinopathy

### Performance of the segmentation model

Segmentation performance was significantly higher in DCP than SCP across all groups (IoU: 96.8 ± 1.2% for DCP vs. 95.2 ± 1.5% for SCP, *P* = 0.0008; DSC: 97.8 ± 0.9% for DCP vs. 97.0 ± 1.1% for SCP, *P* = 0.0007; two-sided paired t-tests). This DCP advantage persisted within DR subgroups (healthy control, NPDR, PDR).

**Fig. 3:**
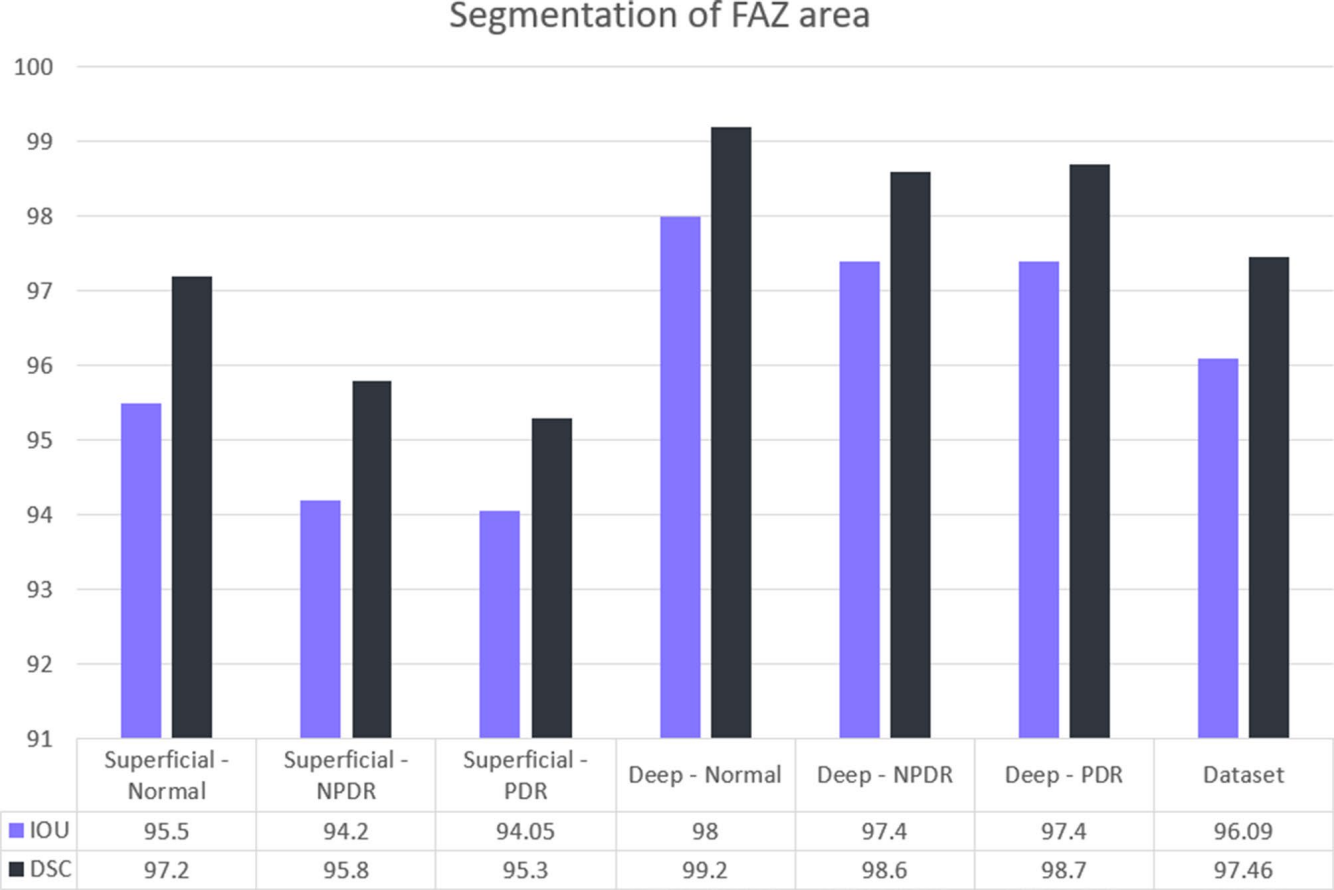
Segmentation accuracy and performance of the deep learning-based model represented by intersection over union (IoU) and dice similarity coefficient (DSC) in both deep capillary plexus (DCP) and superficial capillary plexus (SCP) layers across normal, non-proliferative diabetic retinopathy (NPDR), and proliferative diabetic retinopathy (PDR) categories. Statistics. Segmentation performance of the DCP vs. SCP was compared within eyes using two-sided paired t-tests for both IoU and DSC, overall and by DR subgroup (Normal, NPDR, PDR). IoU *P*-values: Total 0.0008; Normal 0.003; NPDR 0.0009; PDR 0.0006. DSC *P*-values: Total 0.0007; Normal 0.002; NPDR 0.0008; PDR 0.0005

Fig. 3 depicts the segmentation model’s evaluation metrics. Overall, the model achieved an IoU of 96.1% and a DSC of 97.5% on the whole dataset. Both IoU and DSC were significantly higher in the DCP compared to the FAZ segmentation values in the SCP. Using OCTA images of DCP, FAZ segmentation in PDR and NPDR, as well as normal eyes, was done with an IoU and DSC of more than 97%, with higher values belonging to the normal eyes and lower rates yielded from eyes with PDR.

**Fig. 4:**
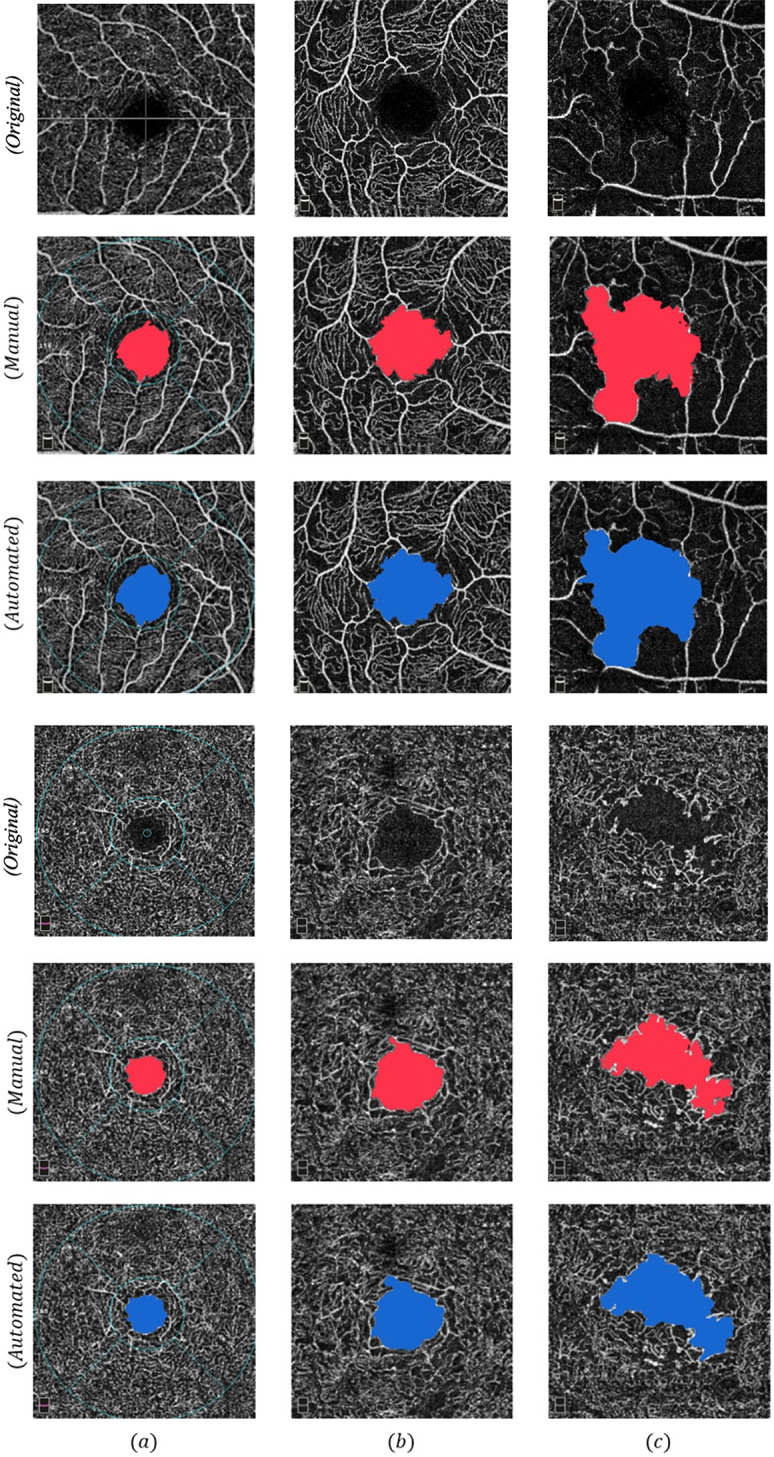
Original images with no segmentation, manual, and automatic segmentation results are shown for the superficial and deep layers across three different classes: (**a**) healthy control, (**b**) non-proliferative diabetic retinopathy (NPDR), and (**c**) proliferative diabetic retinopathy (PDR). The first three rows represent the foveal avascular zone (FAZ) in the superficial capillary plexus (SCP) layer, while the next three rows correspond to the FAZ in the deep capillary plexus (DCP) layer. In the healthy control group, the Early Treatment Diabetic Retinopathy Study (ETDRS) grid overlay was retained in the original OCTA outputs; however, the FAZ region was entirely contained within the central 1 mm circle, and the grid lines did not interfere with FAZ boundary delineation or segmentation performance

Fig. 4 illustrates the visualization results of the model for FAZ segmentation compared to the primary manual segmentation across the three classifications. The model showed satisfactory capability in delineating boundaries of cases with high irregularity.

### Binary classification: normal vs. diseased

**Fig. 5:**
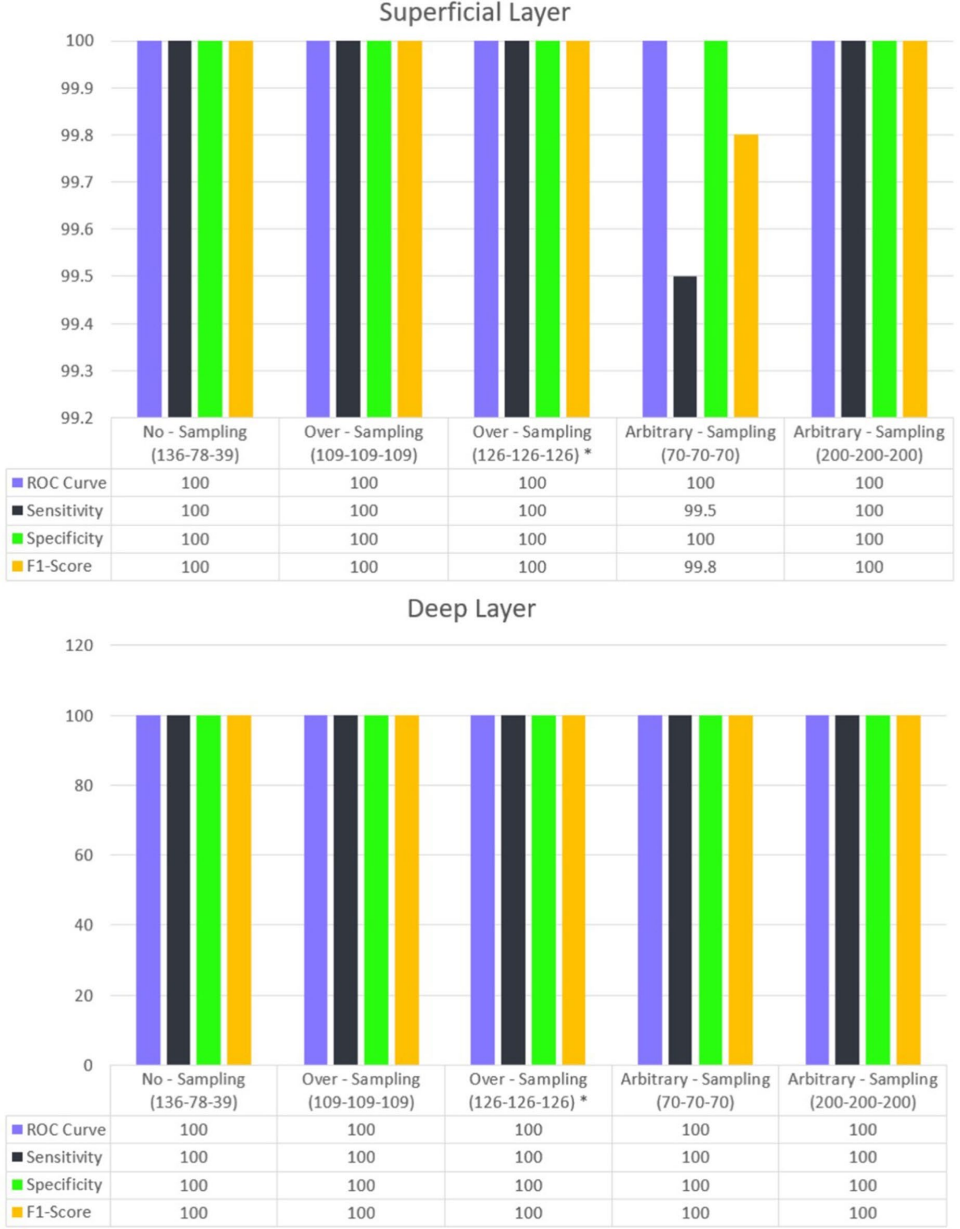
Performance of the binary Classification in different models in the superficial and deep layers. In all examples, the test data from each class is separated by 20% of the total sample size of that class. Only in the example marked with the * symbol is the test data separated by an equal number from each class

Both in the superficial and deep layers, using FAZ for the classification model achieved a perfect score of 100% across all metrics for differentiating between normal eyes from eyes with NPDR or PDR. (Fig. 5) Other accuracy metric values and information for each sample and approach are found in Online Resource 2.

### Three-class classification: normal, NPDR, and PDR

**Fig. 6:**
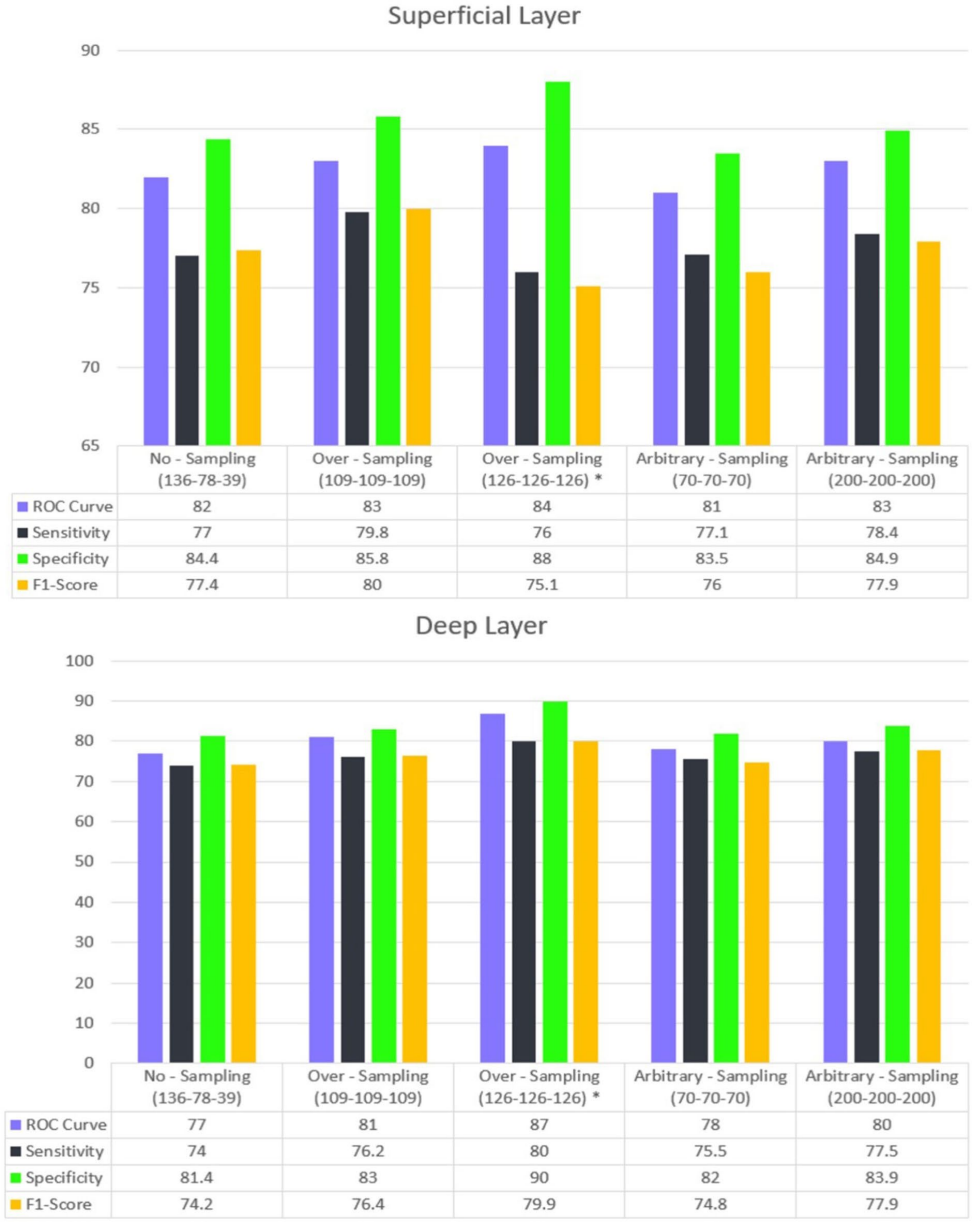
Classification performance across three categories in the superficial and deep layers. In all examples, the test data from each class is separated by 20% of the total sample size of that class. Only in the example marked with the * symbol is the test data separated by an equal number from each class

Overall, the classification results for the superficial layer were superior compared to the deep layer in terms of sensitivity, specificity, F1-score, and AUC. (Fig. 6)

Using FAZ derived from SCP in the original dataset with no sampling method, the model achieved an AUC of 82%, a sensitivity of 77%, a specificity of 84.4%, and an F1-score of 77.4%. The application of oversampling and arbitrary sampling led to a modest yet consistent improvement in classification accuracy. The model’s ability to distinguish between normal, NPDR, and PDR cases was enhanced due to the increased representation of less frequent classes in the training set.

The deep layer classification, while effective, showed a reduced ability to differentiate among the three categories. The AUC for this layer was 77%, with a sensitivity of 74%, a specificity of 81.4%, and an F1-score of 74.2%. The deep layer saw a significant boost in performance following the implementation of oversampling in one of the techniques, with an improvement of approximately 10% compared to the baseline results. (Fig. 6)

To view the other accuracy metric values and information for each sample and approach, please check Online Resource 3.

**Fig. 7:**
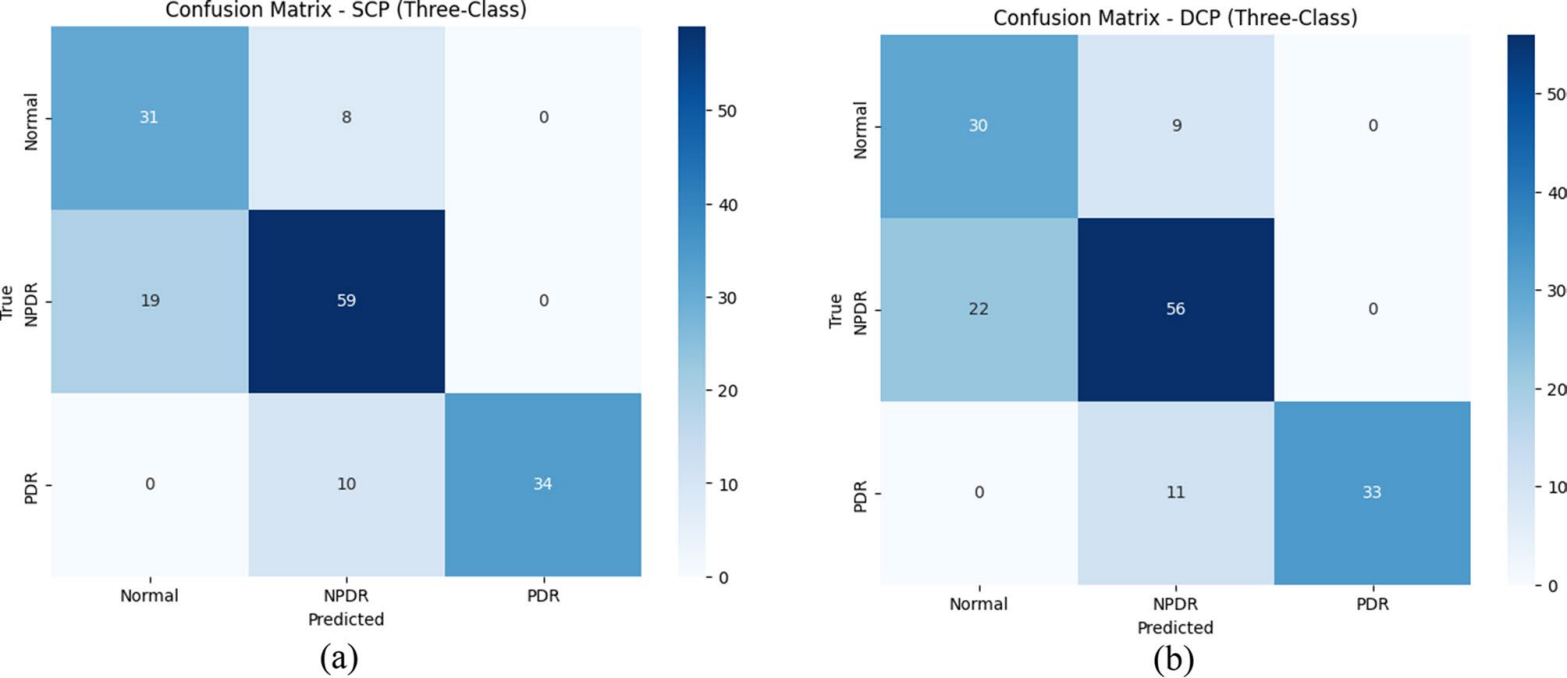
Confusion matrices for three-class classification in (**a**) superficial capillary plexus (SCP) and (**b**) deep capillary plexus (DCP), showing true vs. predicted labels for normal, non-proliferative diabetic retinopathy (NPDR), and proliferative diabetic retinopathy (PDR). Oversampling improved NPDR-PDR differentiation in both layers

**Fig. 8:**
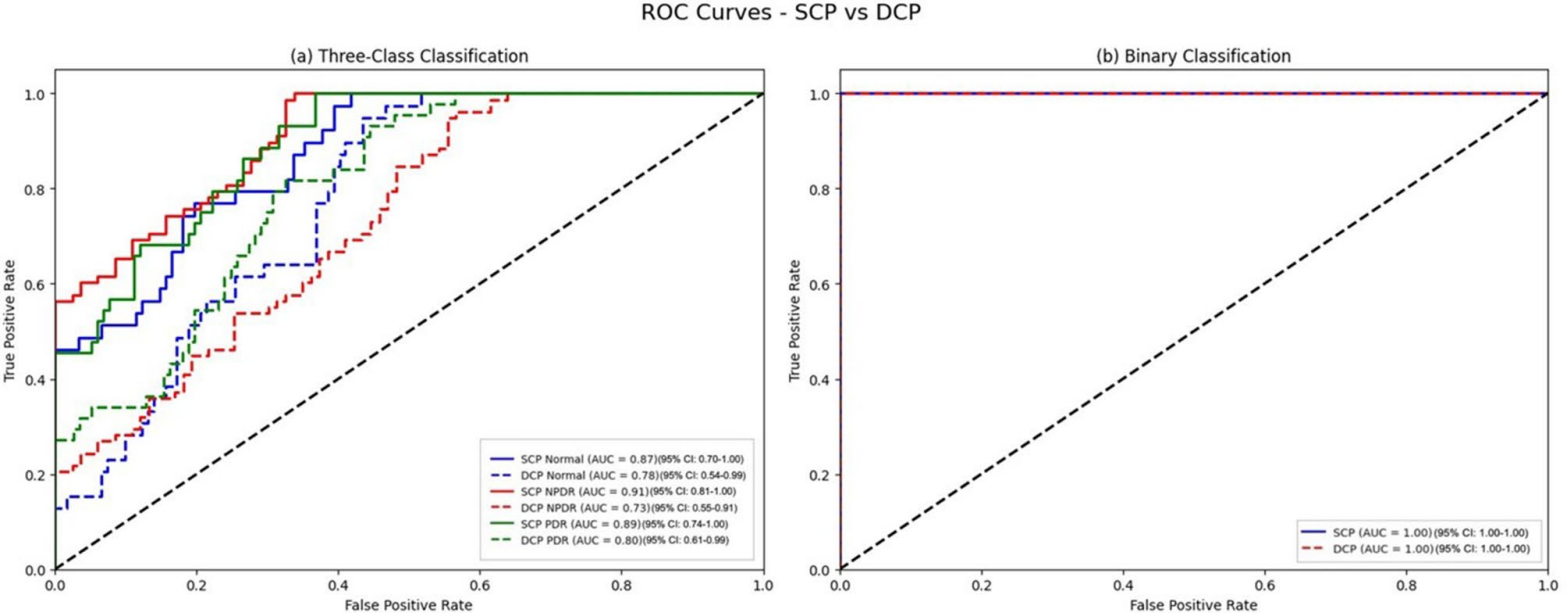
Receiver Operating Characteristic (ROC) curves for superficial capillary plexus (SCP) and deep capillary plexus (DCP). (**a**) Three-class classification (normal, non-proliferative diabetic retinopathy [NPDR], proliferative diabetic retinopathy [PDR]) with average area under the curves (AUC) of 0.84 (SCP) and 0.87 (DCP) using oversampling, demonstrating the model’s discriminative ability across diabetic retinopathy (DR) stages. (**b**) Binary classification (normal vs. DR) with AUCs of 1.00 for both layers, indicating perfect discrimination

Table 3; Figs. 7 and 8 show summaries of segmentation and classification performance.

**Table 3:** Segmentation and classification performance summary

Metric	Layer	Normal	NPDR	PDR	Overall
Segmentation					
IoU (%)	SCP	96.5 ± 1.0	95.8 ± 1.3	94.8 ± 1.6	95.2 ± 1.5
	DCP	97.2 ± 0.8	96.9 ± 1.0	96.4 ± 1.2	96.8 ± 1.2
DSC (%)	SCP	97.8 ± 0.9	97.3 ± 1.0	96.8 ± 1.2	97.0 ± 1.1
	DCP	98.2 ± 0.7	97.9 ± 0.8	97.5 ± 0.9	97.8 ± 0.9
Binary Classification					
AUC (%)	SCP	100	-	-	100
	DCP	100	-	-	100
Sensitivity (%)	SCP	100	-	-	100
	DCP	100	-	-	100
Specificity (%)	SCP	100	-	-	100
	DCP	100	-	-	100
Three-Class Classification					
AUC (%)	SCP	-	-	-	82 (84*)
	DCP	-	-	-	77 (87*)
Sensitivity (%)	SCP	80	75	78	77.4
	DCP	77	72	74	74.2
Specificity (%)	SCP	85	83	86	84.4
	DCP	82	80	83	81.4
F1-Score (%)	SCP	81	76	79	77.4
	DCP	78	73	75	74.2

Note: values with * indicate results with oversampling

AUC: area under the curve, DCP: deep capillary plexus, DSC: Dice similarity coefficient, IoU: Intersection over union, NPDR: non-proliferative diabetic retinopathy, PDR: proliferative diabetic retinopathy, SCP: superficial capillary plexus

## Discussion

In the current study, we presented a novel deep learning-based model for segmenting the FAZ region in OCTA images of patients with NPDR and PDR and healthy controls at both the superficial and deep capillary plexus layers. Additionally, we assessed the utility of the deep learning-derived segmented FAZ regions for differentiating eyes with normal state, NPDR, and PDR in another deep learning framework. The model demonstrated excellent DSC and IoU values of 97.5% and 96.1% on the overall dataset, with the model achieving better results for FAZ segmentation at the DCP layer. For classification, using segmented FAZ regions at both SCP and DCP yielded a 100% AUC, sensitivity, and specificity for distinguishing between normal eyes and DR. However, results pertaining to differentiating between normal eyes, NPDR, and PDR were lower, with an 82% AUC for using FAZ images at the SCP in the original dataset and an AUC of 87% by leveraging sampling methods and FAZ extracted from DCP.

FAZ segmentation in OCTA images holds significant clinical and computational importance in the diagnosis and monitoring of retinal diseases. Despite the compelling reports on the repeatability and reproducibility of FAZ measurement, studies have shown inter-person and even intra-person variability in FAZ morphology, partially attributed to the subjective FAZ segmentation and inter-observer variability [10, 49]. Automating this process using neural networks offers a promising approach to streamline clinical workflows, reducing reliance on manual annotations and thereby saving the time and resources required for manual segmentation. Several reports have evaluated the use of automated models for FAZ segmentation. In studies in which DR was not a particular focus, various segmentation models have been introduced. (Online Resource 1- Table A) In published studies on models developed for FAZ segmentation of OCTA images in patients with no substantial retinal disorder, Guo et al. reported a maximum mean DSC of 97% by leveraging a modified U-Net architecture on the 3 × 3 mm OCTA at the superficial region in patients with low and high myopia [29]. 

It should be highlighted that the challenges associated with segmentation evolve into a more demanding task when combined with the parafoveal capillary dropout surrounding the FAZ in DR and retinal disorders affecting macular perfusion, making the FAZ borders less clear [37]. Consequently, various studies included populations with different retinopathies. Image projection network (IPN), FARGO, Joint-Seg, RPS-Net, and APMFENet are among these models achieving DSC of 88%, 92%, 90%, 91%, and 93% using 6 × 6 mm non-volumetric or volumetric OCTA data at various retinal slabs, respectively [31, 33, 35, 36, 38]. In the current study, we are able to reach higher DSC scores across all groups. However, including a range of retinal disorders rather than only DR as in the present study could be a potential affecting factor in those studies.

Recognizing the practical applications of FAZ segmentation in DR, several studies have used automated models for FAZ segmentation in DR. Lu et al. published one of the first reports, employing generalized gradient vector flow (GGVF) using 3 × 3 mm OCTA images, with Jaccard indices of 83%-87% across various DR categories [28]. Table 4 presents studies using deep learning models for FAZ segmentation in DR. Among the published studies, the reported DSCs ranged from 77% to 94% [39, 40, 42, 43]. In comparison, we were able to obtain a higher DSC in our study. This advantage likely stems from the following factors: we used higher-resolution 3 × 3 mm OCTA with finer vascular detail than 6 × 6 mm, paired a DeepLabv3 + framework and an EfficientNet-B0 backbone augmented with SE blocks to strengthen boundary contrast around the FAZ. A uniform preprocessing further stabilized inputs across layers. Together, these choices yielded more consistent FAZ boundary delineation in challenging DR cases and likely contributed to the observed DSC gains. However, the differences in the methods, including the devices used, the severity of DR in the included participants, exclusion criteria, and the use of DCP, SCP, or full retinal thickness for FAZ segmentation, make direct comparisons between these studies quite challenging. To evaluate our model’s competency, we included OCTA images of patients with PDR, needing more intricate segmentation, for which the DSCs were 95% and 98% in the SCP and DCP layers, respectively.

**Table 4:** Deep learning models for FAZ segmentation using OCTA images in patients with diabetes

Author	Model architecture /design	Retina layer	Quantified/calculated FAZ parameters	Population	OCTA setting	Findings
Mirshahi et al. [39] (2021)	Convolutional neural network based on Detectron 2, compared with manual segmentation (ground truth) and automated measurementa by AngioVue	defined as the inner retinal slab, from the ILM to an offset of 9 μm below the OPL	FAZ area	131 eyes of 88 diabetic patients and 32 eyes of 18 HC	3 × 3 mm OCTA images	Mean DSC of DL model: 0.94 95% limit of agreement between DL and manual measurement in diabetes: 0.063 to 0.095 Correlation coefficient between manual and DL FAZ measurement: 0.962 (excellent)
Guo et al. [40] (2021)	Customized DL model with boundary alignment module (BAM) and boundary supervision module (BSM)	Deep capillary network (between the inner nuclear layer and the outer plexiform layer)	-	80 subjects: 63 HC (12 high myopes and 33 non-high myopes) and 17 DR OCTAGON part of the dataset	3 × 3 mm OCTA images	In DR images: Dice: 0.77 Precision: 0.88 Recall: 0.73
Qiaoyu Li et al. [41] (2022)	A DL U-Net based model for RV and FAZ segmentation complemented by isolated concatenated block (ICB) for information fusion	The maximum projection between ILM and OPL	-	From OCTA-500: 301 OCTA images, 244 labeled HC and 57 labelled DR	3 × 3 mm and 6 × 6 mm OCTA	For FAZ segmentation: Accuracy: 0.92 IoU: 0.84 Overall classification model (RV and FAZ): Accuracy: 0.88 Sensitivity: 0.51 Specificity: 0.96 AUC: 0.92
Qiuzhuo Xu et al. [42] (2022)	Prior guided CNN for 3D FAZ segmentation	-	-	Two datasets, 259 HC eyes, 121 eyes of patients with diabetes mellitus but no DR, 69 eyes with DR (26 with NPDR), 76 eye with high myopia	3 × 3 mm and 6 × 6 mm OCTA	For 6 × 6 mm OCTA: DSC: 0.86 95% HD: 2.7 For 3 × 3 mm OCTA: Dice: 0.94 95% HD: 2.2
Meng et al. [43] (2022)	CNN with U-Net architecture with two attention modules: spatial attention and channel attention blocks	Full-thickness retina	-	Two data sets: 88 eyes designated as diabetic macular ischemia and 32 normal eyes Dataset used in Guo et al. study: 405 OCTA scans of 45 eyes of 45 participants with low and high myopia	6 × 6 mm and 3 × 3 mm OCTA	Results on 1st dataset: DSC: 0.94 Jaccard index: 0.91 Area correlation coefficient: 0.99 Results on 2nd dataset: DSC: 0.98 Jaccard index: 0.96 Area correlation coefficient: 0.95
Totolici et al. [44] (2023)	Four DL models: U-Net U-Net with DenseNet121 U-Net with MobileNetV2 U-Net with VGG16	Full-thickness retina	FAZ area FAZ perimeter FAZ equivalent circle perimeter Acircularity index Angle of FAZ Axis ratio	103 OCT images of 83 subjects augmented to a total of 672 images: 42 images from HC 357 images from DM patients 273 images from patients with both DM and hypertension	6 × 6 mm OCTA	In the DM group and in the best performing model (U-Net + DenseNet121): Accuracy: 0.97 IoU: 0.83 Dice coefficient: 0.90 In the HC and in the best performing model (U-Net + DenseNet121): Accuracy: 0.98 IoU: 0.82 Dice coefficient: 0.90

AI: artificial intelligence, AMD: age-related macular degeneration, AUC: area under the curve, BACC: Balanced accuracy, CNN: convolutional neural network, DM: diabetes mellitus, DL: deep learning, DR: diabetic retinopathy, DSC: Dice similarity coefficient, HC: healthy control, HD: Hausdorff Distance, ICC: intraclass correlation coefficient, IoU: Intersection over union, NPDR: non-proliferative diabetic retinopathy, OCTA: optical coherence tomography angiography, PDR: proliferative diabetic retinopathy, RV: retinal vessel

Our use of 3 × 3 mm scans, offering higher resolution, likely contributed to superior DSC values compared to 6 × 6 mm scans used in prior studies [50]. While wider fields may enhance DR detection [51], our focus on resolution-optimized FAZ segmentation accuracy. Another interesting result of our study was the better segmentation efficacy in the DCP than in the SCP, reflected by higher DSC and IoU. Owing to the vascular network features in DCP and its terminal nature, FAZ is inherently a less distinct region in DCP compared to the SCP. Another source is the higher rate of artifacts in the DCP [52]. This finding may be partially explained by metric sensitivity and boundary complexity. Since the FAZ was larger in the DCP (Table 1), the same boundary error results in a smaller proportional penalty in Dice/IoU. Moreover, while DCP is disrupted earlier in DR and exhibits more pronounced irregularity, SCP changes often manifest later as finer and subtler irregularities. These small-scale features may have increased the perimeter-to-area ratio, making segmentation errors more heavily penalized in overlap metrics for SCP.

Various neural network approaches have been explored for the segmentation of the FAZ region, with CNNs, particularly customized U-Nets [41, 43, 44], being at the forefront due to their effectiveness in extracting spatial hierarchies from image data. In our study, we employed CNN-based architectures. As the segmentation performance of other models mentioned in the method section indicated, the use of pre-trained models such as EfficientNetB0 and DeepLabv3+, along with techniques like the Squeeze-and-Excitation (SE) Block and ASPP, allowed us to enhance the model’s ability to identify and segment the FAZ with high accuracy. This combination of methodologies, paired with the use of large, annotated datasets, ensured reliable segmentation across both the superficial and deep layers of OCTA images. Our results demonstrated that the proposed segmentation framework could serve as a valuable tool in clinical practice, providing precise and consistent FAZ delineation.

In addition to the segmentation efficacy, we also evaluated the potential of generated FAZ segments for DR diagnosis and classification through a novel deep learning-based architecture. Timely diagnosis of DR and optimized screening strategies in eligible patients serve as the principal avenue in effectively preventing and treating DR complications, including loss of vision [53, 54]. In prior studies using FAZ, few reports ascertained the use of FAZ images as input for classification between normal and DR. In one study, they used a U-net-based architecture on both FAZ and retinal vessels and reported an AUC of 92%, sensitivity of 51%, and specificity of 96% in their internal validation dataset [41]. We were able to reach a 100% rate in all of those metrics in our binary classification. Although the state of the DR in the mentioned study is not available, one potential cause of this high rate could be the relatively high number of patients with PDR in our dataset. However, it is worth noting that NPDR patients constituted the majority of our dataset. These high accuracies could have great clinical implications for screening programs, although further external validation is needed.

In one previous study, automatic FAZ-extracted features were used to predict DR classification. They reported that indices like NR300 that consider FAZ variations had the highest correlation with DR severity [28]. We tested whether FAZ images themselves are helpful for differentiating normal, NPDR, and PDR and achieved an AUC of 82% and a specificity of 84% by using SCP FAZ. These numbers were further increased when implementing sampling methods in FAZ at DCP and reached 87% and 90%, respectively. From a clinical perspective, detecting changes in the FAZ shape is invaluable for early diagnosis, prognostic assessment, and treatment planning [17]. Identifying these changes at an early stage allows for timely interventions that can slow or prevent disease progression, underscoring the assistive role of segmentation and analysis of the FAZ region in DR detection.

It is important to note that the perfect performance observed in the binary classification task of the current study likely stems, at least in part, from the relatively higher number of individuals with moderate–severe NPDR compared to mild NPDR, which facilitated the model’s ability to learn distinguishing features between them, in addition to the strength of the network itself. However, this case mix aligns with the setting in which this model was developed based on, highlighting the relevance of adopting a three-class classification framework, since differentiating severe NPDR from PDR is clinically more meaningful than simply distinguishing disease from health, particularly in a tertiary referral population where advanced disease stages predominate. Considering the different management plans in severe NPDR and PDR, and how some could be more invasive in PDR, this makes it even more clinically relevant to differentiate between NPDR cases, with a high prevalence of severe cases, than the PDR cases.

Additionally, the lower performance of the three-class classification compared to the binary setting was also expected. In this framework, the model was trained on fewer images per class and had to separate groups with higher similarity, namely severe NPDR and PDR. Thus, although the three-class task is inherently more demanding and yielded lower performance metrics, it remains the more appropriate and clinically relevant approach for our study setting. While external validation was not performed due to dataset constraints, the model’s robustness across diverse DR severities suggests potential generalizability, pending confirmation in multi-center studies.

In our experiments, we observed that in discriminating between normal, PDR, and NPDR states, the most pronounced differences were observed in the superficial layer of OCTA images. The evidence regarding the utility of the superficial or deep layer is divergent [16, 20, 55]. The higher susceptibility of microvasculature in DCP to ischemic changes has been previously illustrated [11, 56]. Consistently, DCP alterations tend to appear earlier in diabetes, whereas SCP changes become more prominent with advancing disease severity. While this contributes to the incidence of earlier changes in vascular perfusion indices like parafoveal vessel density in DCP compared to SCP, it also leads the SCP to be involved in later stages. This could have led to more pronounced changes in SCP in our cohort, particularly given the higher proportion of severe NPDR and PDR. Since these SCP alterations may appear at more advanced stages, the model may have leveraged this for its higher discriminatory capability. From a practical standpoint, relatively cleaner segmentation and lower artifact burden in SCP may also have favored SCP-derived features [57]. This highlights the importance of the superficial layer for the FAZ classification task, as it can provide distinctive features for identifying disease. However, we also found that the DCP AUC value surpassed that of the SCP when oversampling was applied. This suggests that the class distribution might partially mask DCP’s discriminative signal in smaller datasets; balancing restores that signal. While SCP remained robust in the primary analysis, these findings highlight the complementary role of DCP.

The current framework design can be used as a potential assistive tool for ophthalmologists, integrating into clinical workflows by processing OCTA images to provide automated FAZ segmentation and DR classification. Outputs can be reviewed alongside fundus examinations and FA, reducing manual segmentation time and aiding in early diagnosis.

Despite the promising results, we faced several limitations during both the segmentation and classification stages. The manual annotations also introduced some level of variability, which added complexity to the model’s learning process. We addressed this by evaluating the agreement of the Dice score between the two retinal specialists, with excellent agreement, likely reflecting that both retinal specialists have collaborated for years on similar delineation tasks. This aligns with prior literature reporting near-perfect reproducibility of FAZ measurements, ICCs typically ≥ 0.95–0.99 in SCP/DCP for healthy eyes [58–60], and similarly excellent repeatability in a mixed DR cohort (normal, NPDR, PDR) with ICCs ≥ 0.95–1.00 [61]. While Dice coefficient and ICC capture different aspects of agreement (boundary overlap vs. reproducibility of derived scalars), these data support the reliability of our consensus ground truth for training and evaluation. However, while many studies show excellent FAZ reproducibility, agreement can be modest, particularly for DCP [24], depending on slab definition, device, image quality, and DR spectrum. For instance, a study including diabetic macular edema reported lower agreement rates [62]. In the classification task, the network performed well in differentiating between normal and diseased images. The higher proportion of moderate to severe NPDR in our cohort, resulting from the more advanced case mix typical of a tertiary referral center and thus not a balanced representation of the DR spectrum in a community setting, may have contributed to the perfect discrimination observed in the binary classification. However, distinguishing between NPDR and PDR remained a challenge. These two stages of DR are often characterized by subtle differences in vascular changes, making them harder to differentiate based on FAZ shape alone. The model showed an acceptable accuracy for classifications, but the subtleties in the deep layer images still posed challenges. Moreover, the deep layer images required more computational effort and time from the system to process, with the oversampling techniques providing a significant boost in accuracy, increasing performance by approximately 10. Future research should explore longitudinal FAZ changes to predict DR progression, validate the model across multiple OCTA devices, and integrate FAZ segmentation with vessel density and perfusion metrics for comprehensive DR assessment. We did not include a whole-OCTA classifier in this feasibility phase as a comparison benchmark, since it would introduce non-FAZ cues (e.g., vessel density and non-perfusion areas) and our current dataset was insufficient to support such a more complex task. The use of a single OCTA device, removing eyes with other simultaneous retinal pathologies, and its cross-sectional design are among the other limitations of the current study. While we did not evaluate classifier performance using manual FAZ segmentations as input; however, the very high DSC and IoU between automated and expert masks suggest that any residual gap is likely small, though it cannot be fully excluded. Additionally, the absence of data on diabetes duration and HbA1c levels limits our ability to assess their influence on FAZ morphology and DR severity, potentially affecting the model’s applicability to diverse diabetic populations. Moreover, there have been previous reports on the effect of age and sex on FAZ. However, there were no meaningful differences between the age and sex of the three groups across the study, substantially reducing, though not eliminating, the risk that demographic imbalance impacted our results. Finally, this study is a single-center feasibility investigation with a limited sample size, which constrains generalizability. While uniform acquisition reduced confounding and facilitated method development, external validation across multiple centers and OCTA devices is still needed.

## Conclusion

This study described a comprehensive approach to the segmentation and classification of OCTA images, focusing on the FAZ in both superficial and deep layers and highlighting its clinical importance. By employing advanced neural network architectures such as DeepLabv3 + enhanced with EfficientNetB0 and SE blocks, we achieved highly accurate FAZ segmentation, demonstrating strong agreement with manual annotations by ophthalmologists. These results establish our method as a reliable tool for identifying the FAZ region in OCTA images, providing critical insights into retinal health. In the classification phase, we utilized transfer learning with GoogLeNet to categorize images into two distinct modes: binary classification (normal vs. diseased) and three-class classification (normal, NPDR, and PDR). Our findings revealed that the FAZ shape in the superficial layer provided more distinct features for classification, resulting in higher sensitivity and specificity in distinguishing between normal and diseased states. Despite the challenges in the deep layer, oversampling and augmentation techniques significantly improved classification accuracy, underscoring the importance of addressing data imbalance in deep learning models. Notably, the classification of NPDR and PDR remains a complex task due to subtle morphological differences, requiring further refinement of feature extraction techniques.

We showed that the integration of advanced neural networks and data enhancement techniques demonstrated the potential to overcome challenges associated with data variability and sample size, paving the way for more reliable and efficient diagnostic tools in ophthalmology. These results establish our method as a reliable tool for automated FAZ segmentation and DR classification, potentially enhancing clinical workflows by enabling faster and more accurate diagnosis of DR stages. Future work will focus on further improving the differentiation between NPDR and PDR and expanding the dataset to enhance the model’s generalizability across diverse populations and disease stages for external validation.

## Supplementary Information

Below is the link to the electronic supplementary material.

Supplementary Material 1

Supplementary Material 2

Supplementary Material 3

## Data Availability

The datasets used and analyzed during the current study are available from the corresponding authors upon reasonable request, subject to ethical and privacy constraints.

## Abbreviations

AUC: Area Under the Curve
ASPP: Atrous Spatial Separable Pyramid
CNN: Convolutional Neural Network
DCP: Deep Capillary Plexus
DNN: Deep Neural Networks
DR: Diabetic Retinopathy
DSC: Dice Similarity Coefficient
FA: Fluorescein Angiography
FAZ: Foveal Avascular Zone
IoU: Intersection Over Union
NLM: Non-Local Means
NPDR: Non-Proliferative Diabetic Retinopathy
OCTA: Optical Coherence Tomography Angiography
MDE: Macular Diabetic Edema
MDI: Macular Diabetic Ischemia
PCD: Perifoveal Capillary Density
PDR: Proliferative Diabetic Retinopathy
ReLU: Rectified Linear Unit
SCP: Superficial Capillary Plexus
SE: Squeeze and Excitation

## References

[1] Teo ZL, Tham Y-C, Yu M, et al. Global prevalence of diabetic retinopathy and projection of burden through 2045: systematic review and Meta-analysis. Ophthalmology. 2021;128(11):1580–91. 10.1016/j.ophtha.2021.04.027. 33940045 10.1016/j.ophtha.2021.04.027

[2] Antonetti DA, Klein R, Gardner TW. Diabetic retinopathy. N Engl J Med. 2012;366(13):1227–39. 10.1056/NEJMra1005073. 22455417 10.1056/NEJMra1005073

[3] Cheung CMG, Fawzi A, Teo KY, et al. Diabetic macular ischaemia- a new therapeutic target? Prog Retin Eye Res. 2022;89:101033. 10.1016/j.preteyeres.2021.101033. 34902545 10.1016/j.preteyeres.2021.101033 PMC11268431

[4] Yang Z, Tan TE, Shao Y, Wong TY, Li X. Classification of diabetic retinopathy: Past, present and future. Front Endocrinol (Lausanne). 2022;13:1079217. 10.3389/fendo.2022.1079217. 36589807 10.3389/fendo.2022.1079217 PMC9800497

[5] Gross JG, Glassman AR, Liu D, et al. Five-Year outcomes of panretinal photocoagulation vs intravitreous Ranibizumab for proliferative diabetic retinopathy: A randomized clinical trial. JAMA Ophthalmol. 2018;136(10):1138–48. 10.1001/jamaophthalmol.2018.3255. 30043039 10.1001/jamaophthalmol.2018.3255 PMC6233839

[6] Fong DS, Aiello LP, Ferris FL 3rd, Klein R. Diabetic retinopathy. Diabetes Care. 2004;27(10):2540–53. 10.2337/diacare.27.10.2540. 10.2337/diacare.27.10.2540 15451934

[7] Spaide RF, Fujimoto JG, Waheed NK, Sadda SR, Staurenghi G. Optical coherence tomography angiography. Prog Retin Eye Res. 2018;64:1–55. 10.1016/j.preteyeres.2017.11.003. 29229445 10.1016/j.preteyeres.2017.11.003 PMC6404988

[8] Riazi-Esfahani H, Jafari B, Azimi H, et al. Assessment of area and structural irregularity of retinal layers in diabetic retinopathy using machine learning and image processing techniques. Sci Rep. 2024;14(1):4013. 10.1038/s41598-024-54535-6. 38369610 10.1038/s41598-024-54535-6 PMC10874958

[9] Namvar E, Ahmadieh H, Maleki A, Nowroozzadeh MH. Sensitivity and specificity of optical coherence tomography angiography for diagnosis and classification of diabetic retinopathy; a systematic review and meta-analysis. Eur J Ophthalmol. 2023;33(6):2068–78. 10.1177/11206721231167458. 37013361 10.1177/11206721231167458

[10] Waheed NK, Rosen RB, Jia Y, et al. Optical coherence tomography angiography in diabetic retinopathy. Prog Retin Eye Res. 2023;97:101206. 10.1016/j.preteyeres.2023.101206. 37499857 10.1016/j.preteyeres.2023.101206 PMC11268430

[11] Khalili Pour E, Rezaee K, Azimi H, et al. Automated machine learning-based classification of proliferative and non-proliferative diabetic retinopathy using optical coherence tomography angiography vascular density maps. Graefes Arch Clin Exp Ophthalmol. 2023;261(2):391–9. 10.1007/s00417-022-05818-z. 36050474 10.1007/s00417-022-05818-z

[12] Conrath J, Giorgi R, Raccah D, Ridings B. Foveal avascular zone in diabetic retinopathy: quantitative vs qualitative assessment. Eye. 2005;19(3):322–6. 10.1038/sj.eye.6701456. 15258601 10.1038/sj.eye.6701456

[13] Tang FY, Ng DS, Lam A, et al. Determinants of quantitative optical coherence tomography angiography metrics in patients with diabetes. Sci Rep. 2017;7(1):2575. 10.1038/s41598-017-02767-0. 28566760 10.1038/s41598-017-02767-0 PMC5451475

[14] Mirshahi R, Riazi-Esfahani H, Khalili Pour E, et al. Differentiating features of OCT angiography in diabetic macular edema. Sci Rep. 2021;11(1):23398. 10.1038/s41598-021-02859-y. 34862410 10.1038/s41598-021-02859-y PMC8642537

[15] Kim K, Kim ES, Yu S-Y. Optical coherence tomography angiography analysis of foveal microvascular changes and inner retinal layer thinning in patients with diabetes. Br J Ophthalmol. 2018;102(9):1226. 10.1136/bjophthalmol-2017-311149. 29259019 10.1136/bjophthalmol-2017-311149

[16] Ashraf M, Nesper PL, Jampol LM, Yu F, Fawzi AA. Statistical model of optical coherence tomography angiography parameters that correlate with severity of diabetic retinopathy. Investig Ophthalmol Vis Sci. 2018;59(10):4292–8. 10.1167/iovs.18-24142. 30167660 10.1167/iovs.18-24142 PMC6110573

[17] Takase N, Nozaki M, Kato A, ENLARGEMENT OF FOVEAL AVASCULAR ZONE IN DIABETIC EYES EVALUATED BY EN FACE OPTICAL COHERENCE TOMOGRAPHY ANGIOGRAPHY, et al. Retina. 2015;35(11):2377–83. 10.1097/iae.0000000000000849. 26457396 10.1097/IAE.0000000000000849

[18] Tsai ASH, Jordan-Yu JM, Gan ATL, et al. Diabetic macular ischemia: influence of optical coherence tomography angiography parameters on changes in functional outcomes over one year. Invest Ophthalmol Vis Sci. 2021;62(1):9. 10.1167/iovs.62.1.9. 33404598 10.1167/iovs.62.1.9 PMC7794267

[19] Yang D, Tang Z, Ran A, et al. Assessment of parafoveal diabetic macular ischemia on optical coherence tomography angiography images to predict diabetic retinal disease progression and visual acuity deterioration. JAMA Ophthalmol. 2023;141(7):641–9. 10.1001/jamaophthalmol.2023.1821. 37227703 10.1001/jamaophthalmol.2023.1821 PMC10214181

[20] Sun Z, Tang F, Wong R, et al. OCT angiography metrics predict progression of diabetic retinopathy and development of diabetic macular edema: A prospective study. Ophthalmology. 2019;126(12):1675–84. 10.1016/j.ophtha.2019.06.016. 31358386 10.1016/j.ophtha.2019.06.016

[21] Custo Greig E, Brigell M, Cao F, et al. Macular and peripapillary optical coherence tomography angiography metrics predict progression in diabetic retinopathy: A Sub-analysis of TIME-2b study data. Am J Ophthalmol. 2020;219:66–76. 10.1016/j.ajo.2020.06.009. 32574773 10.1016/j.ajo.2020.06.009

[22] Coscas F, Sellam A, Glacet-Bernard A, et al. Normative data for vascular density in superficial and deep capillary plexuses of healthy adults assessed by optical coherence tomography angiography. Invest Ophthalmol Vis Sci. 2016;57(9):Oct211–23. 10.1167/iovs.15-18793. 27409475 10.1167/iovs.15-18793

[23] Linderman RE, Muthiah MN, Omoba SB, et al. Variability of foveal avascular zone metrics derived from optical coherence tomography angiography images. Translational Vis Sci Technol. 2018;7(5):20. 10.1167/tvst.7.5.20. 10.1167/tvst.7.5.20 PMC6166903 30280005

[24] Shahlaee A, Pefkianaki M, Hsu J, Ho AC. Measurement of foveal avascular zone dimensions and its reliability in healthy eyes using optical coherence tomography angiography. Am J Ophthalmol. 2016;161:50–5.e1. 10.1016/j.ajo.2015.09.026. 26423672 10.1016/j.ajo.2015.09.026

[25] Carpineto P, Mastropasqua R, Marchini G, et al. Reproducibility and repeatability of foveal avascular zone measurements in healthy subjects by optical coherence tomography angiography. Br J Ophthalmol. 2016;100(5):671. 10.1136/bjophthalmol-2015-307330. 26377414 10.1136/bjophthalmol-2015-307330

[26] Pereira B, Faria R, Domingues C, et al. Foveal avascular zone area measurement in diabetic patients: Superficial, deep or combined retinal vascular complex? Microvasc Res. 2025;157:104743. 10.1016/j.mvr.2024.104743. 39260680 10.1016/j.mvr.2024.104743

[27] Eladawi N, Elmogy M, Khalifa F, et al. Early diabetic retinopathy diagnosis based on local retinal blood vessel analysis in optical coherence tomography angiography (OCTA) images. Med Phys. 2018;45(10):4582–99. 10.1002/mp.13142. 30144102 10.1002/mp.13142

[28] Lu Y, Simonett JM, Wang J, et al. Evaluation of automatically quantified foveal avascular zone metrics for diagnosis of diabetic retinopathy using optical coherence tomography angiography. Invest Ophthalmol Vis Sci. 2018;59(6):2212–21. 10.1167/iovs.17-23498. 29715365 10.1167/iovs.17-23498 PMC5958306

[29] Guo M, Zhao M, Cheong AMY, et al. Automatic quantification of superficial foveal avascular zone in optical coherence tomography angiography implemented with deep learning. Vis Comput Ind Biomed Art. 2019;2(1):21. 10.1186/s42492-019-0031-8. 32240395 10.1186/s42492-019-0031-8 PMC7099561

[30] Díaz M, Novo J, Cutrín P, et al. Automatic segmentation of the foveal avascular zone in ophthalmological OCT-A images. PLoS ONE. 2019;14(2):e0212364. 10.1371/journal.pone.0212364. 30794594 10.1371/journal.pone.0212364 PMC6386246

[31] Li M, Chen Y, Ji Z, et al. Image projection network: 3D to 2D image segmentation in OCTA images. IEEE Trans Med Imaging. 2020;39(11):3343–54. 10.1109/TMI.2020.2992244. 32365023 10.1109/TMI.2020.2992244

[32] Carmona EJ, Díaz M, Novo J, Ortega M. Modeling, Localization, and segmentation of the foveal avascular zone on retinal OCT-Angiography images. IEEE Access. 2020;8:152223–38. 10.1109/ACCESS.2020.3017440.

[33] Peng L, Lin L, Cheng P, Wang Z, Tang X, editors. FARGO: A Joint Framework for FAZ and RV Segmentation from OCTA Images. Ophthalmic Medical Image Analysis; 2021 2021//; Cham: Springer International Publishing.

[34] Liu J, Yan S, Lu N, et al. Automatic segmentation of foveal avascular zone based on adaptive watershed algorithm in retinal optical coherence tomography angiography images. J Innovative Opt Health Sci. 2021;15(01):2242001. 10.1142/S1793545822420019.

[35] Hu K, Jiang S, Zhang Y, Li X, Gao X. Joint-Seg: treat foveal avascular zone and retinal vessel segmentation in OCTA images as a joint task. IEEE Trans Instrum Meas. 2022;71:1–13. 10.1109/TIM.2022.3193188.

[36] Li W, Zhang H, Li F, Wang L. RPS-Net: an effective retinal image projection segmentation network for retinal vessels and foveal avascular zone based on OCTA data. Med Phys. 2022;49(6):3830–44. 10.1002/mp.15608. 35297061 10.1002/mp.15608

[37] Khan A, Hao J, Dong Z, Li J. Adaptive Deep Clustering Network for Retinal Blood Vessel and Foveal Avascular Zone Segmentation. Applied Sciences [Internet]. 2023; 13(20).

[38] Quan X, Hou G, Yin W, Zhang H. A multi-modal and multi-stage fusion enhancement network for segmentation based on OCT and OCTA images. Inform Fusion. 2025;113:102594. 10.1016/j.inffus.2024.102594.

[39] Mirshahi R, Anvari P, Riazi-Esfahani H, et al. Foveal avascular zone segmentation in optical coherence tomography angiography images using a deep learning approach. Sci Rep. 2021;11(1):1031. 10.1038/s41598-020-80058-x. 33441825 10.1038/s41598-020-80058-x PMC7806603

[40] Guo M, Zhao M, Cheong AMY, et al. Can deep learning improve the automatic segmentation of deep foveal avascular zone in optical coherence tomography angiography? Biomed Signal Process Control. 2021;66:102456. 10.1016/j.bspc.2021.102456.

[41] Li Q, Zhu XR, Sun G, et al. Diagnosing diabetic retinopathy in OCTA images based on multilevel information fusion using a deep learning framework. Comput Math Methods Med. 2022;2022:4316507. 10.1155/2022/4316507. 35966243 10.1155/2022/4316507 PMC9371870

[42] Xu Q, Li M, Pan N, Chen Q, Zhang W. Priors-guided convolutional neural network for 3D foveal avascular zone segmentation. Opt Express. 2022;30(9):14723–36. 10.1364/oe.452208. 35473210 10.1364/OE.452208

[43] Meng Y, Lan H, Hu Y, et al. Application of improved U-Net convolutional neural network for automatic quantification of the foveal avascular zone in diabetic macular ischemia. J Diabetes Res. 2022;2022:4612554. 10.1155/2022/4612554. 35257013 10.1155/2022/4612554 PMC8898103

[44] Totolici G, Miron M, Culea-Florescu A-L. Automatic segmentation and statistical analysis of the foveal avascular zone. Technologies. 2024;12(12):235.

[45] Chen L-C, Zhu Y, Papandreou G, Schroff F, Adam H, editors. Encoder-Decoder with atrous separable Convolution for semantic image Segmentation. Computer Vision – ECCV 2018; 2018 2018//; Cham: Springer International Publishing.

[46] Tan M, Le Q, EfficientNet. Rethinking Model Scaling for Convolutional Neural Networks. In: Kamalika C, Ruslan S, editors. Proceedings of the 36th International Conference on Machine Learning; Proceedings of Machine Learning Research: PMLR; 2019. p. 6105–14.

[47] Hu J, Shen L, Sun G, editors. Squeeze-and-Excitation Networks. 2018 IEEE/CVF Conference on Computer Vision and Pattern Recognition; 2018 18–23 June 2018.

[48] Chawla NV, Bowyer KW, Hall LO, Kegelmeyer WP. SMOTE: synthetic minority over-sampling technique. J Artif Intell Res. 2002;16:321–57. 10.48550/arXiv.1106.1813.

[49] Laotaweerungsawat S, Psaras C, Haq Z, Liu X, Stewart JM. Racial and ethnic differences in foveal avascular zone in diabetic and nondiabetic eyes revealed by optical coherence tomography angiography. PLoS ONE. 2021;16(10):e0258848. 10.1371/journal.pone.0258848. 34679118 10.1371/journal.pone.0258848 PMC8535464

[50] Ho J, Dans K, You Q, Nudleman ED, Freeman WR, COMPARISON OF 3 MM × 3 MM VERSUS 6 MM × 6 MM OPTICAL COHERENCE TOMOGRAPHY ANGIOGRAPHY SCAN SIZES IN THE EVALUATION OF NON-PROLIFERATIVE DIABETIC RETINOPATHY. Retina. 2019;39(2):259–64. 10.1097/iae.0000000000001951. 29190249 10.1097/IAE.0000000000001951 PMC5963959

[51] Zhu Y, Cui Y, Wang JC, et al. Different scan protocols affect the detection rates of diabetic retinopathy lesions by Wide-Field Swept-Source optical coherence tomography angiography. Am J Ophthalmol. 2020;215:72–80. 10.1016/j.ajo.2020.03.004. 32205122 10.1016/j.ajo.2020.03.004

[52] Lavia C, Bonnin S, Maule M, et al. VESSEL DENSITY OF SUPERFICIAL, INTERMEDIATE, AND DEEP CAPILLARY PLEXUSES USING OPTICAL COHERENCE TOMOGRAPHY ANGIOGRAPHY. Retina. 2019;39(2):247–58. 10.1097/iae.0000000000002413. 30550527 10.1097/IAE.0000000000002413 PMC6358199

[53] Moshfeghi AA, Khurana RN, Moini H, et al. Impact of anti-VEGF treatment on development of proliferative diabetic retinopathy in routine clinical practice. BMC Ophthalmol. 2024;24(1):229. 10.1186/s12886-024-03491-w. 38822279 10.1186/s12886-024-03491-w PMC11140910

[54] Nguyen QD, Moshfeghi AA, Lim JI, et al. Simulation of long-term impact of intravitreal anti-VEGF therapy on patients with severe non-proliferative diabetic retinopathy. BMJ Open Ophthalmol. 2023;8(1):e001190. 10.1136/bmjophth-2022-001190. 37278412 10.1136/bmjophth-2022-001190 PMC10039992

[55] Gill A, Cole ED, Novais EA, et al. Visualization of changes in the foveal avascular zone in both observed and treated diabetic macular edema using optical coherence tomography angiography. Int J Retina Vitreous. 2017;3:19. 10.1186/s40942-017-0074-y. 28642823 10.1186/s40942-017-0074-y PMC5474852

[56] Kim M, Choi SY, Park Y-H. Quantitative analysis of retinal and choroidal microvascular changes in patients with diabetes. Sci Rep. 2018;8(1):12146. 10.1038/s41598-018-30699-w. 30108264 10.1038/s41598-018-30699-w PMC6092390

[57] Ong JX, Kwan CC, Cicinelli MV, Fawzi AA. Superficial capillary perfusion on optical coherence tomography angiography differentiates moderate and severe nonproliferative diabetic retinopathy. PLoS ONE. 2020;15(10):e0240064. 10.1371/journal.pone.0240064. 33091032 10.1371/journal.pone.0240064 PMC7580912

[58] Mihailovic N, Brand C, Lahme L, et al. Repeatability, reproducibility and agreement of foveal avascular zone measurements using three different optical coherence tomography angiography devices. PLoS ONE. 2018;13(10):e0206045. 10.1371/journal.pone.0206045. 30335839 10.1371/journal.pone.0206045 PMC6193722

[59] Shiihara H, Sakamoto T, Yamashita T, et al. Reproducibility and differences in area of foveal avascular zone measured by three different optical coherence tomographic angiography instruments. Sci Rep. 2017;7(1):9853. 10.1038/s41598-017-09255-5. 28851930 10.1038/s41598-017-09255-5 PMC5575252

[60] Sato R, Kunikata H, Asano T, et al. Quantitative analysis of the macula with optical coherence tomography angiography in normal Japanese subjects: the Taiwa study. Sci Rep. 2019;9(1):8875. 10.1038/s41598-019-45336-3. 31221998 10.1038/s41598-019-45336-3 PMC6586606

[61] Lynch G, Romo JSA, Linderman R, et al. Within-subject assessment of foveal avascular zone Enlargement in different stages of diabetic retinopathy using En face OCT reflectance and OCT angiography. Biomed Opt Express. 2018;9(12):5982–96. 10.1364/boe.9.005982. 31065407 10.1364/BOE.9.005982 PMC6491024

[62] Braham IZ, Kaouel H, Boukari M, et al. Optical coherence tomography angiography analysis of microvascular abnormalities and vessel density in treatment-naïve eyes with diabetic macular edema. BMC Ophthalmol. 2022;22(1):418. 10.1186/s12886-022-02632-3. 36329416 10.1186/s12886-022-02632-3 PMC9632091

