# Supplementary material for: Automated FAZ segmentation and diabetic retinopathy classification using OCTA images
Source: BMC Ophthalmol. 2025 Oct 28;25:602. doi: 10.1186/s12886-025-04473-2 (PMC12560293; doi:10.1186/s12886-025-04473-2)
Supplement: Supplementary file 2 — Supplementary Material 2 [file 12886_2025_4473_MOESM2_ESM.pdf]

Figure A:

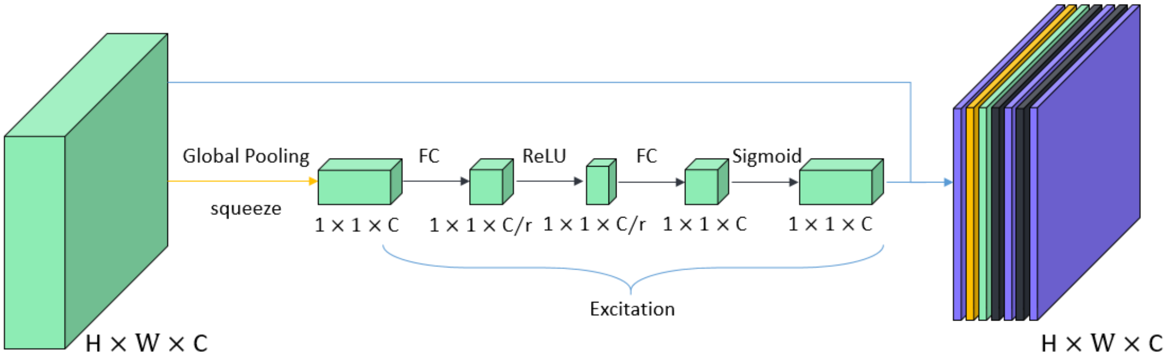

Supplementary Figure A: Architecture of the Squeeze-and-Excitation Network (SENet). The orange arrow denotes the squeeze stage, the blue arrow indicates the scale stage, and the black arrow signifies the excitation stage.

Figure B:

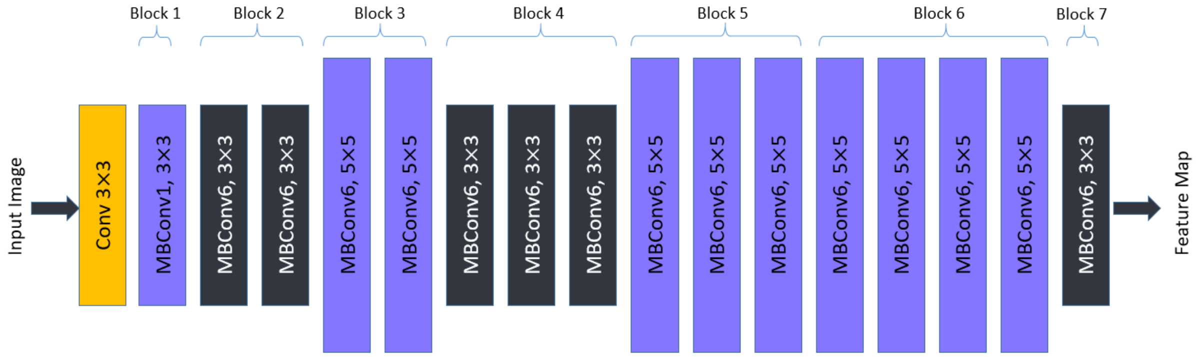

Supplementary Figure B: Architecture of EfficientNet-B0

Figure C:

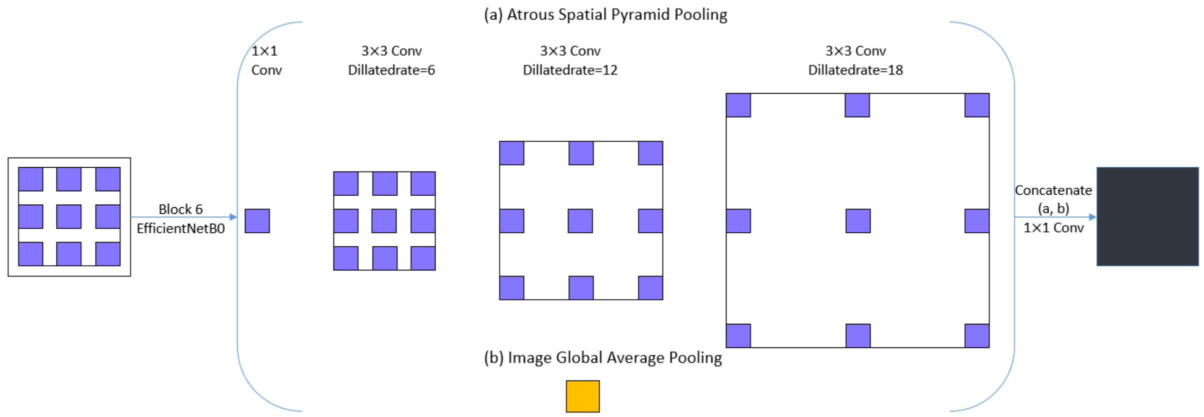

Supplementary Figure C: Structure of the ASPP module used in Deeplabv3+SE. This module consists of two stages, including (a) Atrous convolution and (b) Image Pooling, and produces the final output using a convolution layer after concatenating the feature maps.

The mathematical representation of the ASPP module is given by the formula:

$$y[i] = \sum_{k} x[i + r\,, k]w[k],$$

In this equation, the sampling steps required to process the input feature map are determined by the corresponding dilation rate r. Here, x denotes the input signal, while w represents the filter. When r=1, the standard basic convolution reduces to a specific instance of separable Atrous convolution.

ASPP block enables smaller fields of view, which are crucial for precise region of interest (ROI) localization and effective contextual understanding. Moreover, the implementation of zeros between filter values ensures that the increase in parameters or computations remains minimal, thereby optimizing the overall efficiency of the network. By integrating the ASPP block, we enhance the model's ability to perform semantic segmentation with greater accuracy and efficiency.

The configuration of the ASPP blocks utilized in this study includes four distinct expansion factors: [1, 6, 12, 18], each paired with corresponding kernel sizes of [1, 3, 3, 3]. This setup allows the network to analyze features across multiple scales, thereby enhancing its capability to accurately identify and differentiate the various dimensions of the FAZ. Following the application of these ASPP modules, the outputs were combined through concatenation using a 1 × 1 normal convolution. This concatenation process not only integrates the multi-scale information from the different branches of the ASPP block but also preserves the richness of the feature representations.

Table A. Automated models for FAZ segmentation using OCTA

Author	Model architecture /design	Retina layer	Quantified/calculated FAZ parameters	Population	OCTA Setting	Findings
Eladawi et al.[27] (2018)	DR-CAD (diabetic retinopathy computer-aided diagnosis): Joint Markov-Gibbs random field (MGRF) stochastic model for FAZ segmentation and support vector machine (SVM) based on three estimated features from FAZ: width of the FAZ, blood vessel calibre and vessel density	Superficial and deep retinal layers	-	105 subjects (23 HC and 82 mild DR)	6 × 6 mm OCTA	Average accuracy of the segmentation stage for both superficial and deep layers in HC and DR cases:<br>DSC: 0.95<br>Absolute vessels area difference: 6.9<br>AUC 0.95<br><br>Classification performance of SVM model on three mentioned parameters:<br>Accuracy: 0.94<br>Sensitivity: 0.98<br>Specificity: 0.87<br>AUC: 0.92<br>DSC: 0.96
Lu et al.[28] (2018)	No AI model was used. Generalized gradient vector flow (GGVF) snake model: the region growing algorithm extracted the initial FAZ, and then applied the morphological operator and the Snake model to obtain the final FAZ segmentation.	Inner retina (defined as the slab from the inner limiting membrane to the upper boundary of the outer nuclear layer)	FAZ area<br>Acircularity index<br>STD4<br>NR300	19 HC eyes and 66 diabetic eyes divided into 3 groups:<br>no retinopathy (n =16), mild to moderate NPDR (n = 22), severe NPDR or PDR (n = 28)	3 × 3 mm OCTA, generated by maximum projection of the inner retinal slab.	Jaccard index:<br>0.87 for HC,<br>0.86 for Diabetes without DR<br>0.89 for mild to moderate NPDR<br>0.83 for severe NPDR or PDR<br><br>NR300 had the best sensitivity and correlation with DR severity
Guo et al.[29] (2019)	DL inspired by U-Net	Superficial retina layer	FAZ area	405 OCTA scans of 45 eyes of 45	3 × 3 mm OCTA of with	Maximum mean DSC: 0.97

		(designated as sFAZ)		participants with low and high myopia	robustness and alteration to brightness and contrast (B/C) variations in the images	All DSCs in various groups >0.96
Díaz et al.[30] (2019)	No AI model was used. Image processing based on vascularity edge identification and region growing algorithm	Superficial and deep layer	-	Validation population: OCTAGON dataset: 144 health and 69 diabetic OCTA images	3 × 3 mm and 6 × 6 mm OCTA	Mean Jaccard indexes:<br>In 3-3 mm^2 OCTA:<br>0.83 for superficial<br>0.69 deep layer<br><br>In 6 × 6 mm^2:<br>0.75 for superficial<br>0.64 for deep layer
Mingchao Li et al.[31] (2020)	IPN (Image projection network): Using projection learning modules (PLM) for 3D to 2D images segmentation of RV and FAZ in OCTA	-	-	316 OCTA volumes and corresponding OCT volumes from 293 subjects with various categories: HC, DR, AMD and ...	6 × 6 mm OCTA	For FAZ segmentation:<br>Dice: 0.88<br>Jaccard index: 0.81<br>BACC: 0.94<br>Precision: 0.89<br>Recall: 0.89
Carmona et al.[32] (2020)	Automated image processing techniques (not artificial intelligence), including generation of FAZ normality models, localization and segmentation	Superficial and deep layer	-	OCTAGON dataset:<br>First subset includes 213 OCT-A images (144 HCs and 69 with DR)	3 × 3 mm and 6 × 6 mm OCTA	For DR segmentation:<br>For 6 × 6 mm OCTA in superficial retinal layer:<br>Dice: 0.84<br>Jaccard index: 0.73<br>HD: 0.15<br>For 6 × 6 mm OCTA in deep retinal layer:<br>Dice: 0.83<br>Jaccard index: 0.72<br>95% HD: 0.20<br><br>For 3 × 3 mm OCTA in superficial retinal layer:<br>Dice: 0.86

						Jaccard index: 0.76<br>HD: 0.14<br>For 3 × 3 mm OCTA in deep retinal layer:<br>Dice: 0.82<br>Jaccard index: 0.71<br>HD: 0.17
Peng et al.[33] (2021)	FARGO: DL model for FAZ and RV segmentation:<br>Using a ResNeSt-based U-Net structure combined with RV segmentation as an auxiliary task for FAZ segmentation<br>Spatial attention module and channel attention module joint fusion	-	-	OCTA-500 dataset, including images of HC, DR, age-related macular degeneration and choroidal neovascularizatio n	3 × 3 mm and 6 × 6 mm OCTA	For 6 × 6 mm OCTA:<br>Dice: 0.92<br>Jaccard index: 0.87<br>95% HD: 7.7<br>Average Symmetric Surface Distance (ASSD): 1.5<br><br>For 3 × 3 mm OCTA:<br>Dice: 0.98<br>Jaccard index: 0.96<br>95% HD: 3.1<br>Average Symmetric Surface Distance (ASSD): 0.4
Liu et al.[34] (2022)	Adaptive watershed algorithm<br>Using detection of the length of the “dams" between different regions and the threshold being adjusted adaptively according to the maximum inscribed circle radius of FAZ	Superficial and deep layers	-	33 HC and 25 patients with DR:<br>132 healthy retinal images and 50 DR images	3 × 3 mm OCTA	Correlation coefficient between automatic segmentation and manual segmentation (three physicians):<br>For healthy images >0.94<br>For DR >0.92<br>For low quality images >0.90
Hu et al.[35] (2022)	Joint-Seg (joint segmentation network):<br>Simultaneous FAZ and RV segmentation using DL model using joint encoding block (JEB),	Superficial and deep retinal layer	-	Five datasets:<br>OCTA-500 (training and test)<br>ROSE (test)	3 × 3 mm and 6 × 6 mm OCTA	In OCTA-500 dataset:<br>For 6 × 6 mm OCTA:<br>Dice coefficient: 0.90<br>Jaccard index: 0.84<br>Sensitivity:0.92<br>Specificity: 0.99

	feature adaptive filter (FAF), feature alignment decoder block (FADB) and multiscale soft fusion module (MSFM)			OCTAGON (test)<br>OCTA-25K (test)<br>sFAZ (used by Duo et al) (test)		For 3 × 3 mm OCTA:<br>Dice coefficient: 0.98<br>Jaccard index: 0.96<br>Sensitivity:0.98<br>Specificity: 0.99<br><br>In four other datasets (range):<br>Dice coefficient: 0.72-0.92<br>Jaccard index: 0.60-0.85<br>Sensitivity:0.81-0.98<br>Specificity: 0.98-.0.99
Weisheng Li et al.[36] (2022)	RPS-Net: 3D and 2D data for RV and FAZ by:<br>Two parallel projection paths for simultaneous extraction,<br>A dual-way projection learning module (DPLM),<br>Integration of the features by U-shaped network	-	-	OCTA-500, including images of HC, DR, age-related macular degeneration and choroidal neovascularization	Volumetric 3D data and projection maps of 3 × 3 mm and 6 × 6 mm OCTA	For 6 × 6 mm OCTA:<br>Dice: 0.91<br>Jaccard index: 0.85<br>BACC: 0.95<br><br>For 3 × 3 mm OCTA:<br>Dice: 0.97<br>Jaccard index: 0.95<br>BACC: 0.99
Khan et al.[37] (2023)	Supervised an unsupervised learning based on adaptive segmented deep clustering (ASDC) approach and K-means clustering for simultaneous RV and FAZ segmentation	Between the inner retina's internal limiting membrane and outer plexiform layer	-	Part of OCTA-500 dataset: 300 subjects	6 × 6 mm OCTA scans	The values are for R and FAZ segmentation combined:<br>Accuracy:<br>Unsupervised: 0.89<br>Supervised: 0.96<br><br>Precision:<br>Unsupervised: 0.67<br>Supervised: 0.91<br><br>Recall:<br>Unsupervised:0.79

						Supervised: 0.83<br><br>Dice: 0.86<br>Jaccard index: 0.79
Quan et al.[38] (2025)	APMFENet (Adaptive Projection and Multi stage Fusion Enhancement Network):<br>Turning 3D data of OCT and OCTA into RV and FAZ segmentation in 2D data through Kernel Adaptive Projection Module (KAPM), Volume Fusion Module (VFM), Multi-stage Complementary Fusion Module (MCFM), Multi-modal Differential Fusion Module (MDFM) and three<br>UNet structures and a feature interaction module	-	-	OCTA-500 dataset:<br>500 sample of various categories including images of HC, DR, age-related macular degeneration and choroidal neovascularization	6 × 6 mm and 3 × 3 mm OCTA	Mean IoU: 0.83<br><br>For 6 × 6 mm OCTA:<br>Dice: 0.93<br>Jaccard index: 0.88<br>BACC: 0.96<br><br>For 3 × 3 mm OCTA:<br>Dice: 0.98<br>Jaccard index: 0.96<br>BACC: 0.99

AI: artificial intelligence, AMD: age-related macular degeneration, AUC: area under the curve, BACC: Balanced accuracy, CNN: convolutional neural network, DL: deep learning, DR: diabetic retinopathy, DSC: Dice similarity coefficient, HC: healthy control, HD: Hausdorff Distance, ICC: intraclass correlation coefficient, IoU: Intersection over union, NPDR: non-proliferative diabetic retinopathy, OCTA: optical coherence tomography angiography, PDR: proliferative diabetic retinopathy, RV: retinal vessel

Table B. Key parameters and hyperparameters used in this study for fine-tuning EfficientNetB0.

**Parameter/Hyper parameter**	**Value/Setting**	**Description**
Learning Rate	0.001	Step size for updating weights during training.
Optimizer	ADAM	Optimization algorithm with adaptive learning rates
Batch Size	32	Number of images processed in a single forward/backward pass.
Epochs	100	Number of complete passes through the training dataset.
Input Image Size	352 x 352	Dimensions of input OCTA images.
Pretrained Weights	ImageNet	Initial weights for the EfficientNetB0 architecture.
Loss Function	Binary Cross Entropy	Suitable for binary-class classification problems.
Dropout Rate	0.2	Regularization technique to reduce overfitting.
Max Pooling	2*2	These are used to reduce the spatial dimensions of the feature maps and focus on the most critical features.
Activation function	ReLU	The Rectified Linear Unit is employed across convolutional and other feature extraction layers to introduce non-linearity and enhance learning capacity.
Number of Parameters	~15 million	Total trainable parameters in the fine-tuned EfficientNetB0 model.

Table C. Key parameters and hyperparameters used in this study for fine-tuning GoogLeNet.

**Parameter/Hyper parameter**	**Value/Setting**	**Description**
Learning Rate	0.001	Step size for updating weights during training.
Optimizer	SGDM	Use a Training Options SGDM object to set training options for the stochastic gradient descent with momentum optimizer, including learning rate information, L2 regularization factor, and mini-batch size
Batch Size	50	Number of images processed in a single forward/backward pass.
Epochs	30	Number of complete passes through the training dataset.
Input Image Size	352 x 352	Dimensions of input OCTA images.
Pretrained Weights	ImageNet	Initial weights for the GoogLeNet architecture.
Loss Function	Categorical Cross entropy	Suitable for multi-class classification problems.
Dropout Rate	0.5	Regularization technique to reduce overfitting.
Activation function	Softmax	Used in the final classification layer to output probabilities for the multi-class classification task.
Number of Parameters	~7 million	Total trainable parameters in the fine-tuned GoogLeNet model.
